# Chikungunya virus antagonizes cGAS-STING mediated type-I interferon responses by degrading cGAS

**DOI:** 10.1371/journal.ppat.1008999

**Published:** 2020-10-15

**Authors:** L. G. Webb, J. Veloz, J. Pintado-Silva, T. Zhu, M. V. Rangel, T. Mutetwa, L. Zhang, D. Bernal-Rubio, D. Figueroa, L. Carrau, R. Fenutria, U. Potla, St. P. Reid, J. S. Yount, K. A. Stapleford, S. Aguirre, A. Fernandez-Sesma

**Affiliations:** 1 Department of Microbiology, Icahn School of Medicine at Mount Sinai, New York, NY, United States of America; 2 The Graduate School of Biomedical Sciences at Icahn School of Medicine at Mount Sinai, New York, NY, United States of America; 3 Department of Microbiology, New York University School of Medicine, New York, NY, United States of America; 4 Department of Microbial Infection and Immunity, The Ohio State University, Columbus, OH, United States of America; 5 Infectious Diseases Institute, The Ohio State University, Columbus, OH, United States of America; 6 Department of Pathology & Microbiology, University of Nebraska Medical Center, Omaha, NE, United States of America; 7 Department of Medicine, Division of Infectious Diseases, Icahn School of Medicine at Mount Sinai, New York, NY, United States of America; University of Texas Southwestern Medical Center at Dallas, UNITED STATES

## Abstract

Chikungunya virus (CHIKV) is a mosquito-borne alphavirus known to cause epidemics resulting in predominantly symptomatic infections, which in rare cases cause long term debilitating arthritis and arthralgia. Significant progress has been made in understanding the roles of canonical RNA sensing pathways in the host recognition of CHIKV; however, less is known regarding antagonism of CHIKV by cytosolic DNA sensing pathways like that of cyclic GMP-AMP synthase (cGAS) and Stimulator of Interferon Genes (STING). With the use of cGAS or STING null cells we demonstrate that the pathway restricts CHIKV replication in fibroblasts and immune cells. We show that DNA accumulates in the cytoplasm of infected cells and that CHIKV blocks DNA dependent IFN-β transcription. This antagonism of DNA sensing is via an early autophagy-mediated degradation of cGAS and expression of the CHIKV capsid protein is sufficient to induce cGAS degradation. Furthermore, we identify an interaction of CHIKV nsP1 with STING and map the interaction to 23 residues in the cytosolic loop of the adaptor protein. This interaction stabilizes the viral protein and increases the level of palmitoylated nsP1 in cells. Together, this work supports previous publications highlighting the relevance of the cGAS-STING pathway in the early detection of (+)ssRNA viruses and provides direct evidence that CHIKV interacts with and antagonizes cGAS-STING signaling.

## Author summary

Chikungunya virus (CHIKV) is an emerging vector-borne virus which causes outbreaks that spread within populations quickly. The virus has been shown to be highly susceptible to type-I interferon signaling and can block this antiviral pathway. No work has identified if CHIKV is able to block the induction of type-I IFN by antagonizing cytosolic DNA sensing. Here we show that CHIKV induces the accumulation of DNA in the cytoplasm of infected cells and that the virus blocks cGAS-STING dependent innate immune sensing via degradation of cGAS. This work provides the first description of CHIKV mediated inhibition of DNA dependent innate immune sensing and expands upon our previous works by identifying and characterizing another RNA virus family able to counteract cGAS-STING mediated DNA sensing.

## Introduction

CHIKV is an arbovirus belonging to the genus Alphavirus (Family: *Togaviridae*) which is transmitted primarily by mosquitos of the *Aedes spp*[1, 2]. Approximately 70–90% of CHIKV infections are symptomatic and the hallmark of infection is an acute febrile illness with a sudden high-grade fever as well as debilitating arthritis and arthralgia which can persist, in some infected individuals, for months to years after clearing the infection[3–6]. Since its first characterization in 1952, CHIKV has been noted to cause sporadic and explosive outbreaks resulting in a potentially debilitating range of inflammatory diseases[7–9]. Between 2006–2007, CHIKV was responsible for an outbreak on the island of La Reunion where more than one third of the island’s population was infected in the span of a few months[10]. This outbreak then spread to India, where an estimated 1.3 million infections occurred, Southeast Asia, and ultimately to Italy where the first subtropical autochthonous transmission was established[11, 12].

The CHIKV genome is a positive-sense single stranded RNA (+ssRNA) with a type 0, 5’ methyl-guanosine cap and poly(A) tail, which encodes six structural and four non-structural proteins. After entry and uncoating of the viral nucleic acid, translation of the non-structural proteins occurs. Two polyproteins are produced: either p123 or, if read-through of an opal stop codon takes place, p1234[13–15]. Non-structural protein 1 (nsP1) of CHIKV has been shown to anchor viral replication complexes to cytosolic membranes through both an amphipathic helix as well as through palmitoylation of three cystine (Cys) residues 417–419[16, 17]. Mutations of these residues have been shown for both CHIKV and other alphaviruses to severely hamper viral replication kinetics and decrease pathogenicity in mouse models[18, 19]. Viral structural proteins are then translated from the 26S RNA and processed threaded into the endoplasmic reticulum (ER) with exception of the capsid protein which remains in the cytosol prior to encapsidation of the viral genomic RNA[20].

Innate immune defenses are at the forefront of restricting viral pathogens. Successful replication and subsequent release of viral progeny is largely dependent upon the ability of a virus to evade or inhibit both detection and activation of cellular antiviral responses. CHIKV is no exception, the type-I interferon (IFN) response has been demonstrated to be key in controlling CHIKV infection[21–26] and primary sensing of the virus is via the pattern recognition receptor (PRR) retinoic acid-inducible gene I (RIG-I)-mitochondrial antiviral signaling protein (MAVS) axis[24, 26]. Alternatively to pathogen associated molecular patterns (PAMPs), cell intrinsic molecules released as a result of cellular stress, termed danger associated molecular patterns (DAMPs), are also potent inducers of innate immune responses[27, 28] which lead to the induction of type-I IFNs. Type-I IFNs are a family of cytokines, composed of both IFN alpha (IFN*a*) and IFN*ß*, which are induced though PRR signaling[29]. These cytokines signal in an autocrine and paracrine manner, and are critical in inducing an antiviral state in uninfected cells proximal to infected cells[29]. Antiviral states are induced by transcription and translation of hundreds of interferon stimulated genes (ISGs) as a result of type-I IFN signaling[30]. Previous work has shown that CHIKV nsP2 inhibits IFN mediated signaling[31–33] downstream of type-I IFN production and can inhibit RNA pol II dependent transcription[34]. Meshram *et al*. Identified that mutations within nsP2 alter the production of IFN beta (IFN*ß*)[35]. To date, however, no function has been identified for any CHIKV proteins, in restricting the induction of type-I IFN due to DNA stimuli.

While the canonical view of PRR mediated antiviral responses associates RNA sensing PRRs with RNA based pathogens and DNA sensing PRRs with DNA based pathogens, these systems do not exist in isolation. Recent work has demonstrated a role for cytosolic DNA innate immune sensors in initiating and responding to RNA viral infections and it has been shown that there is a crosstalk present between RIG-I like receptor (RLR) signaling and DNA signaling through the adaptor protein, stimulator of interferon genes (STING)[36, 37]. Specifically, the cyclic GMP-AMP synthase (cGAS)-STING pathway has been shown to restrict both human flaviviruses and coronaviruses and the viruses in-turn have mechanisms to inhibit the pathway[38–46]. Antiviral responses are activated when cGAS binds to double stranded DNA or DNA-RNA hybrids and synthesizes the secondary messenger molecule, 2’-3’ cyclic-guanosine monophosphate (GMP)-adenosine monophosphate (AMP), (cyclic GMP-AMP or cGAMP) which then bind to STING[47, 48]. Upon cGAMP binding, STING dimerizes, translocates to the Trans-Golgi-Network (TGN), where it associates with Tank Binding Kinase 1 (TBK1), which ultimately results in phosphorylation of Interferon Regulatory Factor 3 (IRF3) and induction of type-I IFN transcription[41, 42, 49, 50]. Interestingly, activation of the cGAS-STING pathway with chemical activators or via overexpression has been shown to restrict alphaviruses, including CHIKV[21, 23, 25]; however, no studies have identified direct antagonism of the cGAS-STING pathway by CHIKV. Given the importance of type-I IFN in restricting CHIKV, as well as recent work highlighting the relevance of the cGAS-STING pathway in restricting RNA viruses, we asked whether CHIKV was able to antagonize the cGAS-STING pathway.

## Results

### CHIKV inhibits DNA induced type-I IFN transcription in primary human cells

**Fig 1:**
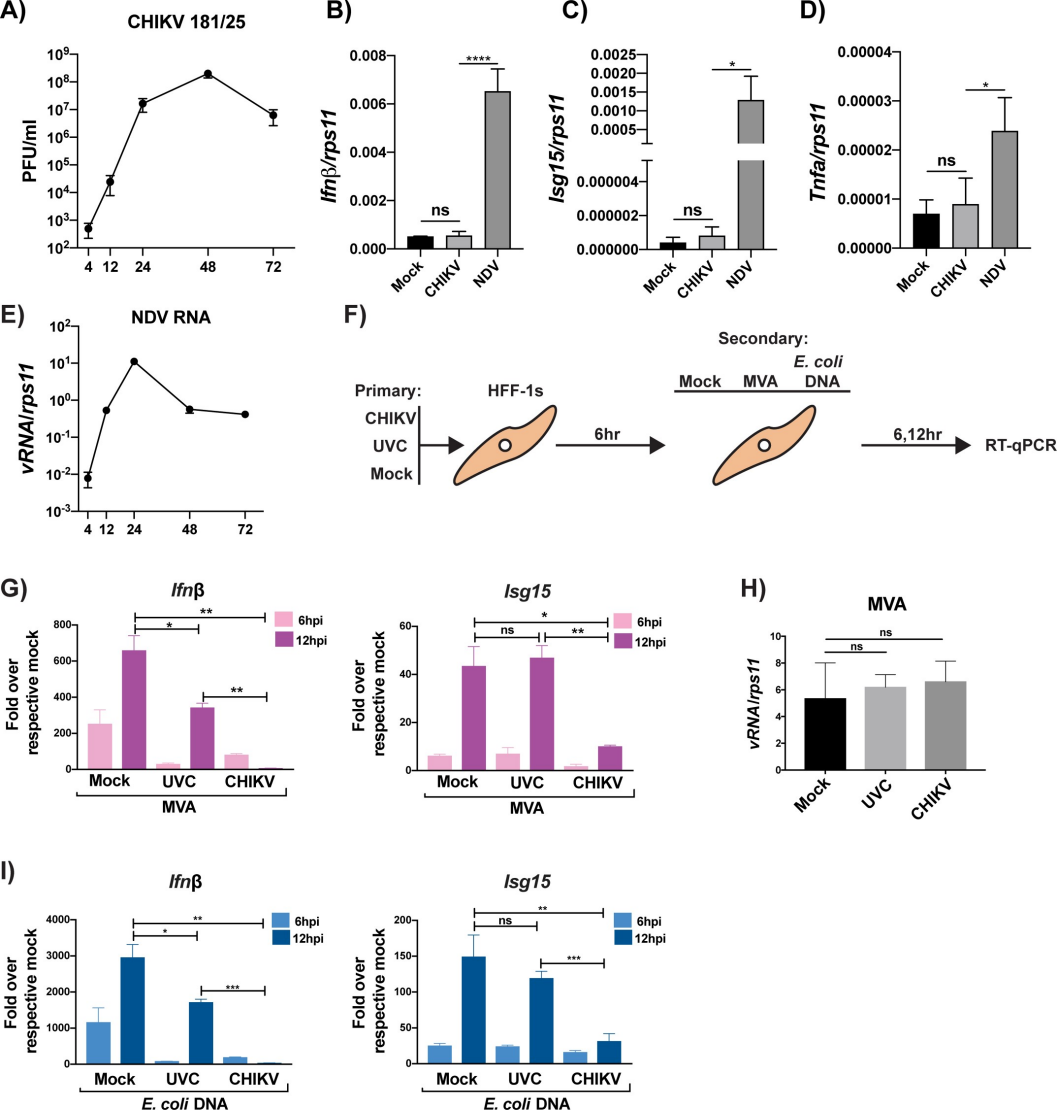
CHIKV inhibits DNA induced type-I Interferon transcription in primary human cells. **(A)** HFF-1s were infected with CHIKV 181/25 (MOI = 0.1) and supernatants were plaqued on BHKs at indicated timepoints. **(B-D)** RT**-**qPCR from CHIKV growth curve in (A) for specified genes (*Ifnb*, *Isg15*, *and Tnfa*) relative to *rps11* at 12 hpi. Data representative of three independent experiments. Data represented as means ± SD (n = 3). Statistical analysis was performed with by a one-way ANOVA with Tukey’s multiple comparisons. **(E)** NDV viral RNA relative to *rps11* from infected HFF-1s (MOI = 0.1) at indicated timepoints. Data represented as means ± SD (n = 3). **(F)** Diagram of coinfection experiments. Briefly, HFF-1s were infected with either mock, UV-inactivated CHIKV 181/25 (UVC), or CHIKV 181/25 at an MOI of 5.0. Infections were allowed to proceed for 6 hrs in order to allow for viral protein expression. **(G & I)** 6 hpi, cells were then treated with mock, MVA at an MOI of 2.0, or E. coli DNA (1ug/2.0X10^5^ cells). 6 and 12 hrs postsecondary treatment, cell lysates were collected. *Ifnß* transcripts were quantified via RT-qPCR for cells stimulated with MVA or E. coli DNA, respectively, while quantification of *Isg15* transcripts was performed as previously stated **(G & I).** Transcripts are represented as “fold over respective mock” E.G. UVC ➔ MVA condition was normalized to UVC ➔ mock condition to determine the relative gene induction resultant from secondary treatment. **(H)** Quantification of MVA transcripts at 12hrs post infection. **(G-I)** Data representative of two independent experiments (n = 3). Data are represented by means ± SD (n = 3). Statistical analysis was done with student’s t tests. Statistical significance represented as follows: ns = not significant, * = p<0.05, ** = p<0.01, *** = p<0.001, **** = p<0.0001.

CHIKV has been shown to replicate well in primary human foreskin fibroblast (HFF-1) cells and fibroblasts are thought to be primary sites of viral amplification upon infection[51]. To better understand the innate immune response induced in primary human cells by CHIKV 181/25, HFF-1s were infected either with CHIKV 181/25 or the paramyxovirus Newcastle disease virus (NDV). NDV, like CHIKV, is sensed via RLRs[28]; however, NDV is an avian pathogen with limited ability to antagonize the induction of the innate immune response in human cells[52, 53]. CHIKV 181/25 replicated to high titers in HFF-1s (Fig 1A) but had a notably muted induction of *Ifn-ß*, *Isg15*, *and TNFa* transcripts at 12 hpi when compared to NDV (Fig 1B–1D). NDV replication was measured in HFF-1s and peaked by 24 hpi (Fig 1E). To better understand if CHIKV 181/25 had similar replication in HFF-1s to other CHIKV isolates, it’s replication and transcriptional profile were compared to a CHIKV isolate of the Indian Ocean lineage (CHIKV-IOL). CHIKV 181/25 replicated better in HFF-1s when compared to CHIKV-IOL but did not induce largely different innate immune responses at the transcriptional level (S1 Fig).

To investigate the ability of CHIKV to block DNA sensing, HFF-1 cells were infected first with infectious or UV-inactivated CHIKV (UVC) and subsequently infected with either Modified Vaccinia Ankara virus (MVA), a DNA virus known to be sensed via cGAS[54], or as an alternative source of cGAS ligand, transfected with *E*. *coli* DNA, respectively (Fig 1F). Surprisingly, CHIKV drastically reduced *Ifn-ß* transcription induced by both a DNA virus (MVA) and a direct cGAS agonist (E. *coli* DNA) (Fig 1G & 1I). A slight reduction in *Ifn-ß* transcripts was observed in the UVC condition (Fig 1G & 1I). This minor inhibition could be mediated by an intrinsic component of the viral nucleocapsid, playing a role in reducing cGAS-STING signaling. Importantly, the initial infections did not alter the ability of MVA to replicate in these cells at 12 hpi, demonstrating that the reduction in innate immune transcripts observed in Fig 1G & 1I is a result of CHIKV-mediated inhibition and not due to a reduction in MVA replication (Fig 1H).

The role of individual CHIKV non-structural proteins in potential inhibition of type-I IFN production was tested using a previously established type-I IFN reporter system[38]. These reporter cells are HEK-293T cells that are stably transduced to express firefly luciferase under the control of an interferon beta (*Ifnβ*) promoter and are deficient for both cGAS and STING (293T-IFNβ-FFluc) (S2A Fig). The ability of CHIKV nsPs to inhibit DNA mediated induction of the *Ifnβ* promotor was assessed by co-expressing the non-structural proteins of CHIKV, Ross Thailand isolate (CHIKV-RT), with cGAS and STING. 36 hrs post transfection, firefly luciferase activity was quantified as a proxy for activity of the *Ifnβ* promoter. Inhibition of DNA dependent *Ifnβ* promoter activity was observed for nsPs 1, 2, and 3 (S2B & S2C Fig). Inhibition of cGAS-STING dependent *Ifnβ* promoter induction by nsPs 1 and 3 was similar to, or greater than, levels of inhibition seen with nsP2 as well as DENV NS2B3, our positive control for inhibition of the pathway[38, 39] (S2B & S2C Fig). To test for CHIKV nsP mediated degradation of STING, nsPs of CHIKV-RT were co-expressed with STING. When STING was co-expressed with the four individual CHIKV-RT nsPs no degradation or cleavage of STING was observed, though a clear cleavage of STING was seen in the positive control, STING co-expressed with DENV NS2B3[39] (S2D Fig). Additionally, nsPs 1–4 of CHIKV-RT were individually expressed with cGAS to look for potential interactions or degradation of the protein. Immunoprecipitation of cGAS pulled down nsP4 of CHIKV-RT; however, no degradation of cGAS was observed in any of the co-expression conditions (S2E Fig). Next, we validated the interaction of CHIKV 181/25 nsP4 with cGAS in the context of infection by over-expression of cGAS in HEK-293T for 24 hrs followed by infection with CHIKV 181/25 and subsequent cGAS immunoprecipitation. Interestingly, no cGAS was observed in the input sample of the infected condition with CHIKV 181/25 (S2F Fig, lane 2). However, when the immunoprecipitation for cGAS was performed, we detected a reduced presence of cGAS, even less than in the positive control, DENV NS2B3, which has already been shown to interact with and degrade cGAS[38] (S2F Fig). To further confirm the interaction between nsP4 and cGAS, HEK-293Ts were transfected with CHIKV-RT nsP4 and seeded on glass-bottom plates for imaging by immunofluorescence. A clear co-localization of cGAS with nsP4 was observed in cells which expressed both proteins (S2G Fig). The lack of cGAS degradation seen in the exogenous expression system when compared to the infected cells shows that although there is a clear interaction of cGAS with nsP4, the degradation of cGAS observed is not mediated by the non-structural proteins of CHIKV, but must require other viral or host factors.

### CHIKV infection induces accumulation of cytosolic DNA in primary human cells

**Fig 2:**
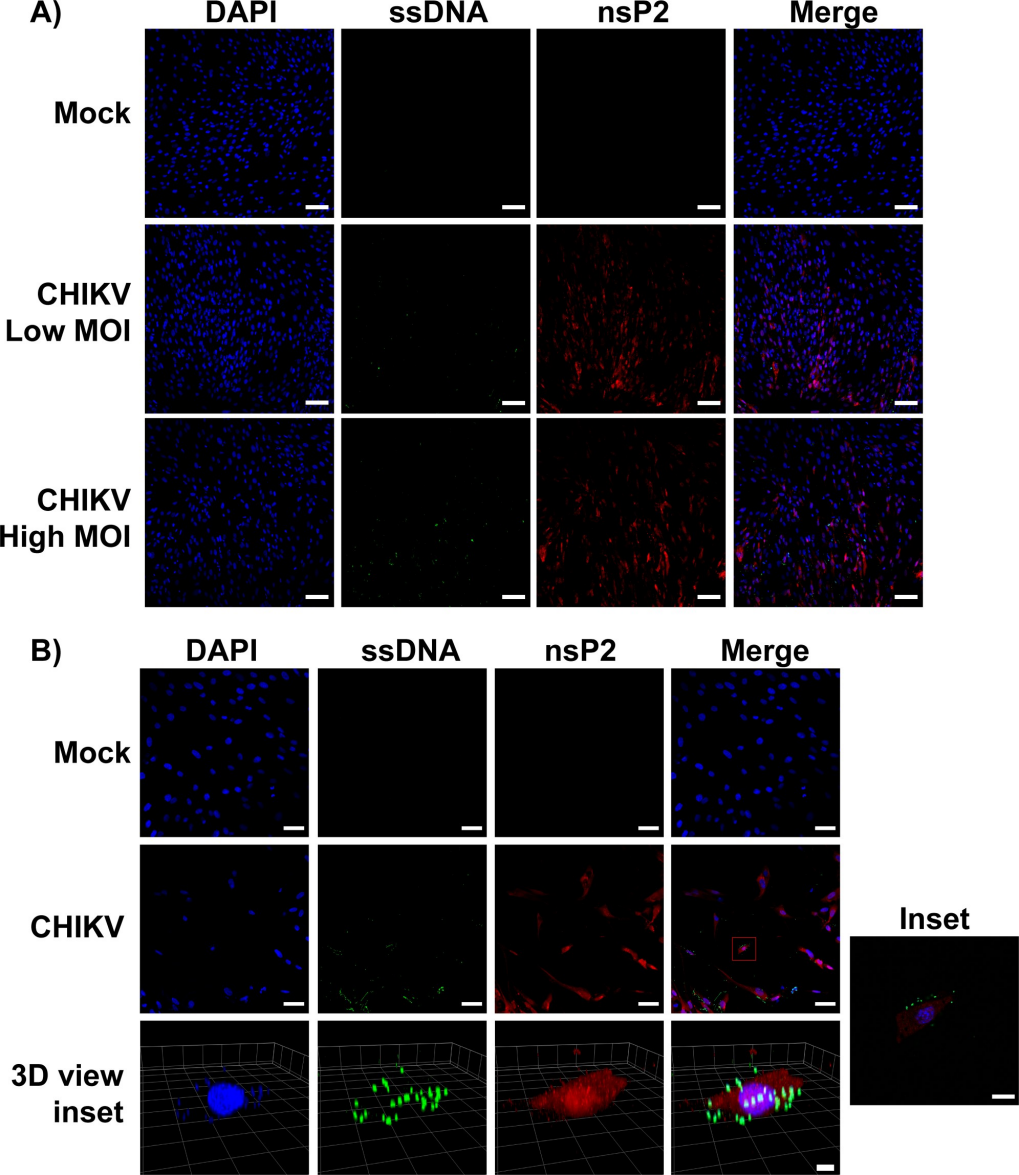
CHIKV infection results in extra-nuclear DNA in primary cells. HFF-1s were infected with either mock or different MOIs of CHIKV 181/25 (low = 1.0, high = 10.0). 24 hpi cells were fixed with 4% formaldehyde and permeabilized with 0.01% triton-X before staining with antibodies specific for nsP2 (red), DNA (green), or DAPI (blue). **(A)** HFF-1s infected at different MOIs of CHIKV were visualized using a Zeiss LSM 880 with Airyscan. Scale bars = 100um. **(B)** HFF-1s infected with CHIKV (MOI = 10.0) for 24hrs. Imaging performed with a Zeiss LSM 880 with Airyscan and 3D inset image was generated via reconstruction of Z-Stacks with Zen Blue software. Scale bars: 50um (2D), 50 Pixels (3D of inset). Data are representative of three independent experiments.

During viral infection, stresses placed upon cells can lead to the release of DNA into the cytoplasm of infected cells, thus leading to the activation of cytosolic DNA sensors, such as cGAS[38, 55]. We previously detected such cytosolic DNA in DENV infected cells via immunofluorescence[38]. To test if CHIKV infection results in the appearance of cytoplasmic DNA in infected cells, HFF-1s were infected with CHIKV 181/25 at low or high multiplicity of infection (MOI) for 24hrs. The cells were then probed with antibodies against nsP2 (viral infection), an anti-DNA antibody, clone 16–19 (Millipore), which binds both double stranded and single stranded DNA (dsDNA/ssDNA), and DAPI which binds dsDNA. During CHIKV infection we observed a distinct accumulation pattern of DNA puncta which were absent in the mock infected condition (Fig 2A). Furthermore, the presence of DNA puncta increased in conjunction with the increase in MOI (Fig 2A). High resolution confocal microscopy of infected cells illustrated the presence of this DNA to be in the cytoplasm (Fig 2B (3D View Inset)).

### cGAS and STING restrict CHIKV infection

**Fig 3:**
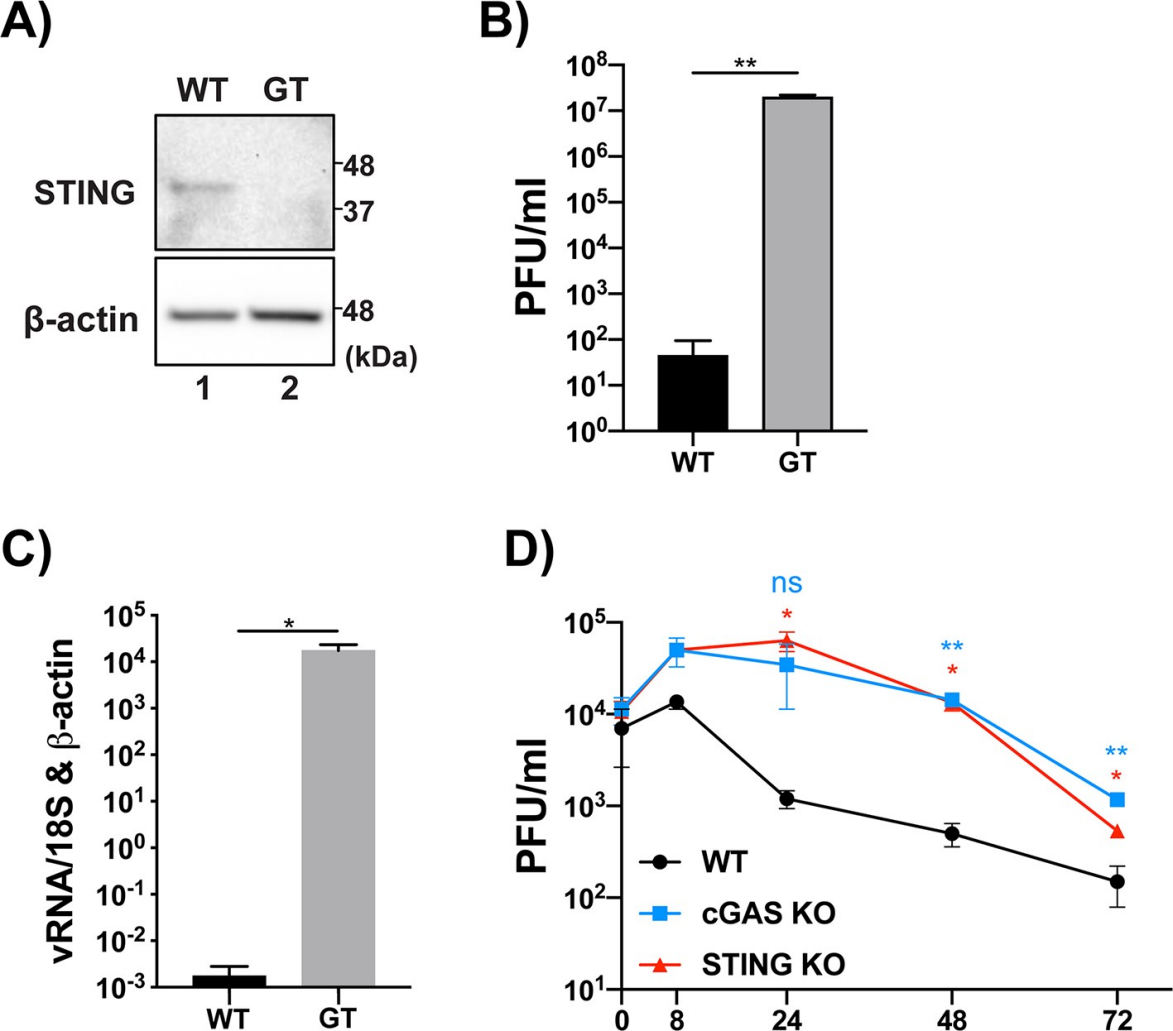
cGAS and STING restrict CHIKV replication and infectious particle release. **(A)** WT or GT MEFs we lysed and analyzed via SDS-PAGE and immunoblotting. **(B)** WT and GT MEFs infected with CHIKV 181/25 (MOI = 0.1) for 16hr. infectious particles in the supernatant were quantified via plaque assay. **(C)** RT-qPCR of viral RNA (*nsp2*) relative to *18S* and *b-actin*. **(A-C)** representative of three independent experiments. Data are represented by means ± SD (n = 3). Statistical analysis was done with student’s t tests. **(D)** WT, cGAS KO, or STING KO RAW 264.7 cells were infected with CHIKV 181/25 (MOI = 0.1) and supernatants were collected at indicated timepoints. Infectious particle release in the supernatant was quantified via plaque assay. Data representative of three independent experiments. Data are represented by means ± SD (n = 3). Statistical analysis was done via two-way ANOVA with Tukey’s multiple comparisons. Statistical significance represented as follows: ns = not significant, * = p<0.05, ** = p<0.01, *** = p<0.001.

Wild type murine embryonic fibroblasts (MEFs) have been shown to be permissive to CHIKV replication[56]. We obtained WT and STING null, *goldenticket* (GT) MEFs[57] (Fig 3A) and infected them with CHIKV 181/25 in order to understand the restriction imposed by STING on CHIKV. By 16hpi there was a 5 log increase in infectious particle release from the GT MEFs when compared to WT (Fig 3B). Viral transcripts were also significantly lower in WT MEFs when compared to their STING null counterparts (Fig 3C) indicating that murine STING serves as a potent restriction factor for CHIKV replication and infectious particle release. Next, commercially available RAW 264.7 cells, a murine macrophage cell line, deficient for cGAS or STING, were infected with CHIKV 181/25. Infectious virus release was quantified from the supernatant of infected cells and revealed a sharp increase in CHIKV replication from both the cGAS and STING null cells when compared to their WT counterpart (Fig 3D). Taken together, these data support previous reports that the cGAS-STING pathway restricts the replication of CHIKV[21, 23, 25] and provides direct evidence that the pathway poses a significant restriction upon viral replication.

### CHIKV infection targets cGAS for immediate degradation upon viral infection

**Fig 4:**
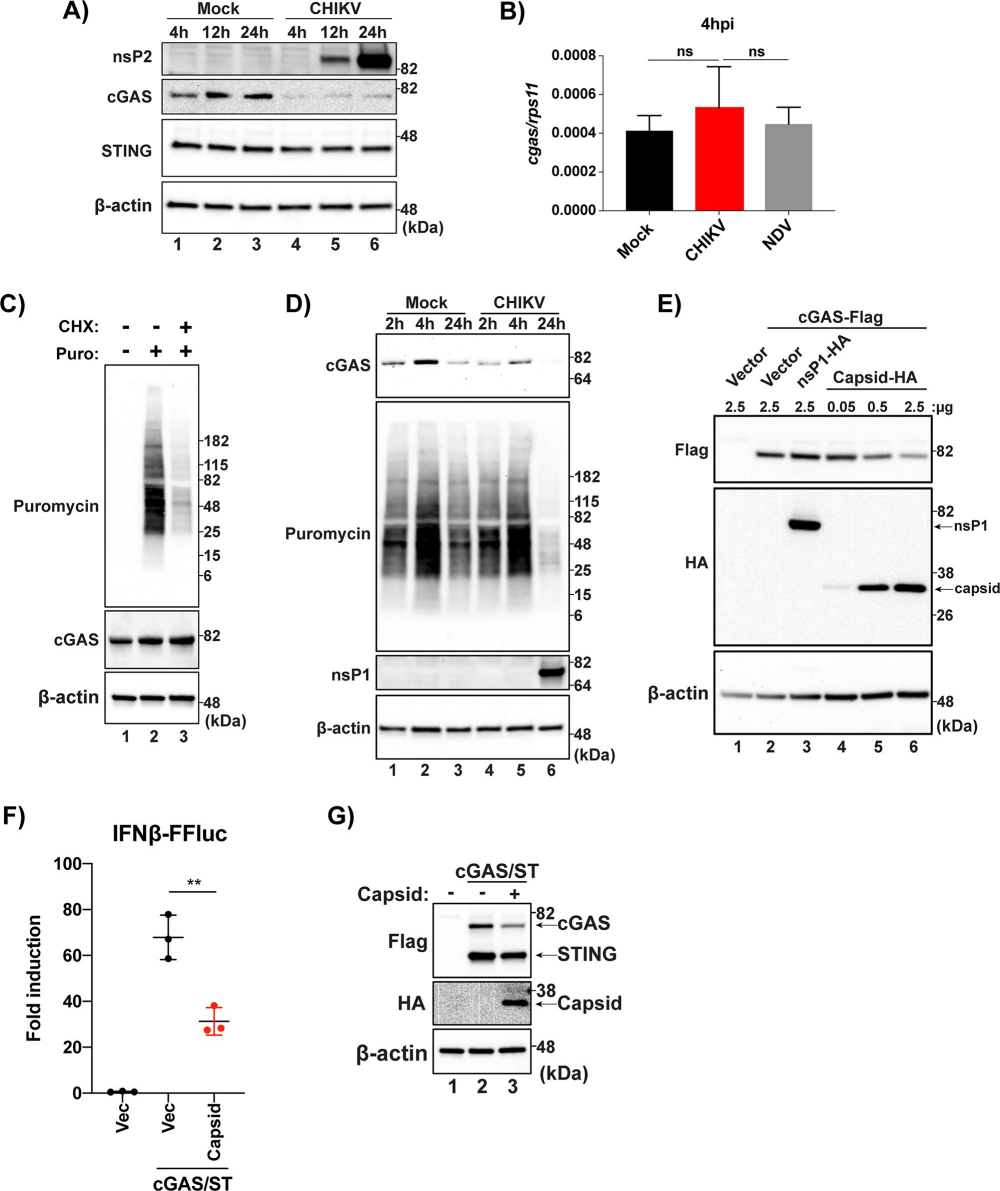
**(A)** HFF-1s were infected with either mock or CHIKV 181/25 at an MOI of 0.1 and cell lysates were collected 4, 12, and 24 hpi. Analysis of protein from mock or CHIKV infected cells was performed via SDS-PAGE followed by immunoblotting for indicated proteins (data representative of three independent experiments). **(B)** RT-qPCR for *cgas* transcripts from HFF-1s infected with CHIKV (MOI 0.1), NDVB1 (MOI 0.1), or mock at 4hpi (data representative of three independent experiments. Data are represented by means ± SD (n = 3). Statistical analysis was done with student’s t tests. **(C)** HFF-1s treated with mock or CHX at 200 μg/ml for 5hrs then treated with mock or puromycin at 10 μg/ml for 15 min. After 15 min puromycin pulse, cells were washed with dPBS and re-fed complete media. Lysates collected and visualized via SDS-PAGE and immunoblotting (data representative of two independent experiments). **(D)** HFF-1s were infected with mock or CHIKV at an MOI of 1.0. 45 min prior to indicated timepoints, cells were pulsed with puromycin as described in **(C).** Lysates were collected and proteins were detected as previously stated (data representative of two independent experiments). **(E)** HEK-293Ts were transfected with indicated constructs at indicated plasmid amounts. Cells were allowed to rest for 24 hr post transfection before lysis and SDS-PAGE/immunoblotting analysis (data are representative of three independent experiments). **(F)** 293T-IFNb-FFluc cells were transfected with empty vector (Vec), cGAS and STING in conjunction with Vec, or cGAS and STING with the capsid of CHIKV 181/25. Cells were allowed to rest for 36hrs before lysis for collection of protein or quantification of luminescence. data are representative of six independent experiments. Data represented as fold induction over Vector alone. Data are represented by means ± SD (n = 3). Statistical analysis was done with student’s t tests (** = p<0.01)). **(G)** Protein input for IFN reporter assay in **(F)**.

To determine the integrity of the cGAS during CHIKV infection, whole cell lysates were collected from HFF-1s at 4, 21, and 24 hpi. Lysates were analyzed via SDS-PAGE and endogenous cGAS and STING were detected via Western blotting (WB). We observed a drastic decrease in cGAS expression as early as 4 hpi, before even viral non-structural proteins were detectable via WB in infected cells, while STING expression was not altered (Fig 4A). Because CHIKV can inhibit cellular transcription, the reduction in cGAS expression over time in CHIKV infected cells could also be due to reduced cGAS transcripts, ultimately leading to reduced newly translated cGAS. mRNA levels of cGAS were quantified and there were no differences in CHIKV infected cells when compared to mock or NDV at 4 hpi (Fig 4B) indicating that a decrease in cGAS transcripts is not responsible for the rapid loss of cGAS expression following CHIKV infection.

Alternatively, cGAS levels could be reduced via global translational inhibition by the viral protein nsP2[32], resulting in a reduction in cGAS protein levels through normal homeostatic protein recycling. To assess if the half-life of cGAS was shorter than the time in which it takes CHIKV infection to result in a loss of cGAS expression, a puromycin pulse-chase was performed. This technique has been shown to be highly sensitive in detecting a snapshot of active translation in cells[58]. HFF-1s were pretreated with mock or cycloheximide (CHX) for 5 hrs to block cellular translation and then “pulsed” for 15 min with puromycin to label newly synthesized proteins after which cells were washed and re-fed with complete media for 45 min. We observed a clear reduction of puromycin incorporation in cells which were treated with CHX for 5 hrs, demonstrating an inhibition of new protein synthesis in treated cells. Although translation had been halted in CHX treated cells for 5 hrs, there was no change in cGAS expression, demonstrating that the half-life of cGAS in HFF-1s is longer than 5hrs (Fig 4C). In order to assess whether viral induced translational shutoff coincides with loss of cGAS expression, CHIKV-mediated translational inhibition was determined by the previously described puromycin pulse-chase method (Fig 4D). In accordance with a previous publication[26], CHIKV mediated translational shutoff was not detectable at 2 or 4 hpi while there was a significant reduction in cGAS expression as early as 2 hpi, when compared to mock (Fig 4D), demonstrating that the loss of cGAS expression observed in infected cells is not due to CHIKV mediated translational inhibition. Interestingly, there is a noticeable increase in translation in both mock and infected cells by 4 hpi suggesting that the stress produced during the process of infection (mock or CHIKV) in HFF-1s stimulates increased translation at early timepoints (Fig 4D, lanes 2 and 5).

As we observed a slight decrease in DNA stimuli-dependent type-I IFN transcripts in the UVC conditions (Fig 1G & 1I) and because the decrease in cGAS occurs very early during the infection, we hypothesized that the viral factor responsible for cGAS degradation is a component of the virion. Upon viral membrane fusion in endosomes, the viral nucleocapsid is exposed in the cytoplasm which is followed by disassembly of the nucleocapsid resulting in the release of the viral genome along with capsid into the cytoplasm of infected cells. To determine if CHIKV capsid was sufficient to result in a degradation of cGAS, the viral protein was co-expressed with cGAS in increasing amounts. A dose dependent cGAS degradation was observed when co-expressed with capsid and this reduction was specific, as no reduction in cGAS expression was observed when the innate immune sensor was co-expressed with another viral protein, nsP1 (Fig 2E). Capsid co-expression was also sufficient to inhibit cGAS-STING mediated induction of a type-I IFN reporter, indicating a functional inhibition of the innate immune sensing pathway (Fig 4F & 4G). These data indicate an immediate restriction of the cGAS-STING pathway by CHIKV via degradation of cGAS. This degradation is independent of viral transcriptional and translational shutoff and is mediated by the viral capsid protein.

### Degradation of cGAS during CHIKV infection occurs via an ATG7 dependent autophagic mechanism

**Fig 5:**
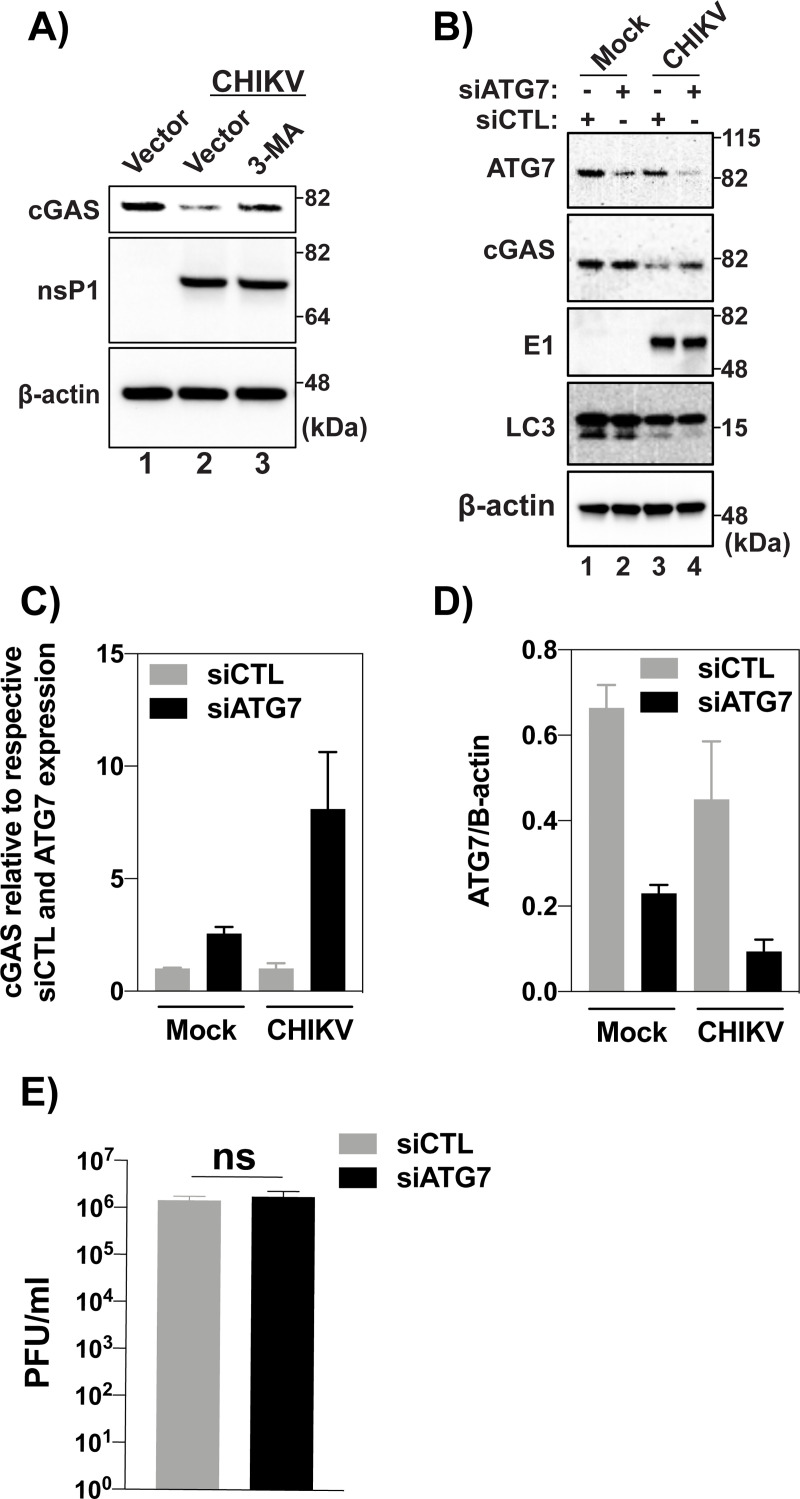
**(A)** 293Ts transfected with 200 ng cGAS for 16hrs then infected with CHIKV at an MOI of 10.0. Infections were allowed to proceed for 10 hrs before treatment with 3-MA at 5 mM. Cells were allowed to rest for 8 hrs after drug treatment and were lysed. Lysates were visualized via SDS-PAGE followed by immunoblotting with indicated antibodies. (Data representative of three independent experiments). **(B)** U2OS cells were transfected with 10 nm siRNA (control or ATG7) for 48 hrs and infected 24 hpt with CHIKV (MOI = 10) or mock infection. Cells were collected and lysed 24 hpi. Analysis by western blot using indicated antibodies. **(C)** Fold change in cGAS expression relative to respective non-targeting siRNA controls and ATG7 expression via densitometry analysis of western blot bands in figure (n = 2) (B). **(D)** Quantification of ATG7 knockdown in U2OS cells from western shown in figure **(B)** (n = 2)**. (E)** Plaque assay of supernatants collected from infected U2OS cells at 24hrs post infection (n = 3) Data are represented as means ± SD. (B-E) Data representative of two independent experiments. Statistical analysis was done with student’s t test.

During infection, CHIKV is known to induce autophagy which has a proviral effect on replication[59]. Previous work from our group demonstrated an autophagy-mediated degradation of cGAS during DENV infection, in order to prevent the activation of the cGAS-STING pathway by mis-localized mtDNA [38]. We hypothesized that the cGAS degradation observed in CHIKV infection might also occur via autophagy. To test this, HEK-293Ts were transfected with cGAS before treatment with a commonly used chemical inhibitor of autophagy, 3-methyladenine (3-MA)[38, 60–62] and later infected with CHIKV 181/25. Treatment with 3-MA was able to rescue expression of cGAS in infected cells, indicating a role for autophagy in cGAS degradation during CHIKV infection (Fig 5A).

Confirmation that autophagy participates in cGAS degradation during CHIKV infection was assayed via knockdown of autophagy related protein 7 (ATG7), a critical component in the formation of phagophores[63]. Knockdown of ATG7 was able to rescue cGAS expression in infected cells, but did not have an appreciable effect in non-infected cells (Fig 5B). Importantly, knockdown of ATG7 did not result in an accumulation of the lower molecular weight band of LC3, demonstrating a functional inhibition of the process of autophagy in the *siAtg7* conditions (Fig 5B). Upon quantitative analysis of protein expression, ATG7 knockdown did increase expression of cGAS in mock-infected cells by 2.6 fold, when compared to a non-targeting control, indicating that ATG7 participates in regulating homeostatic levels of cGAS expression (Fig 5C). In CHIKV infected conditions, however, there was a striking 8.1 fold increase in cGAS expression due to ATG7 knockdown, confirming that autophagy modulates cGAS expression during CHIKV infection (Fig 5C). Reduction in ATG7 expression when compared to respective non-targeting controls were 65.4% (mock) and 79.2% (CHIKV), respectively (Fig 5D). No difference in viral infectious particle release was observed between *siAtg7* or *siCTL*, indicating that the increase in cGAS expression was not due to reduced viral replication (Fig 5E). These data demonstrate a critical role of autophagy in CHIKV dependent cGAS degradation and expand our previous work with DENV, suggesting a conserved virus-host interplay across different class IV RNA viruses.

### CHIKV nsP1 interacts with STING resulting in signaling inhibition and viral protein stabilization

**Fig 6:**
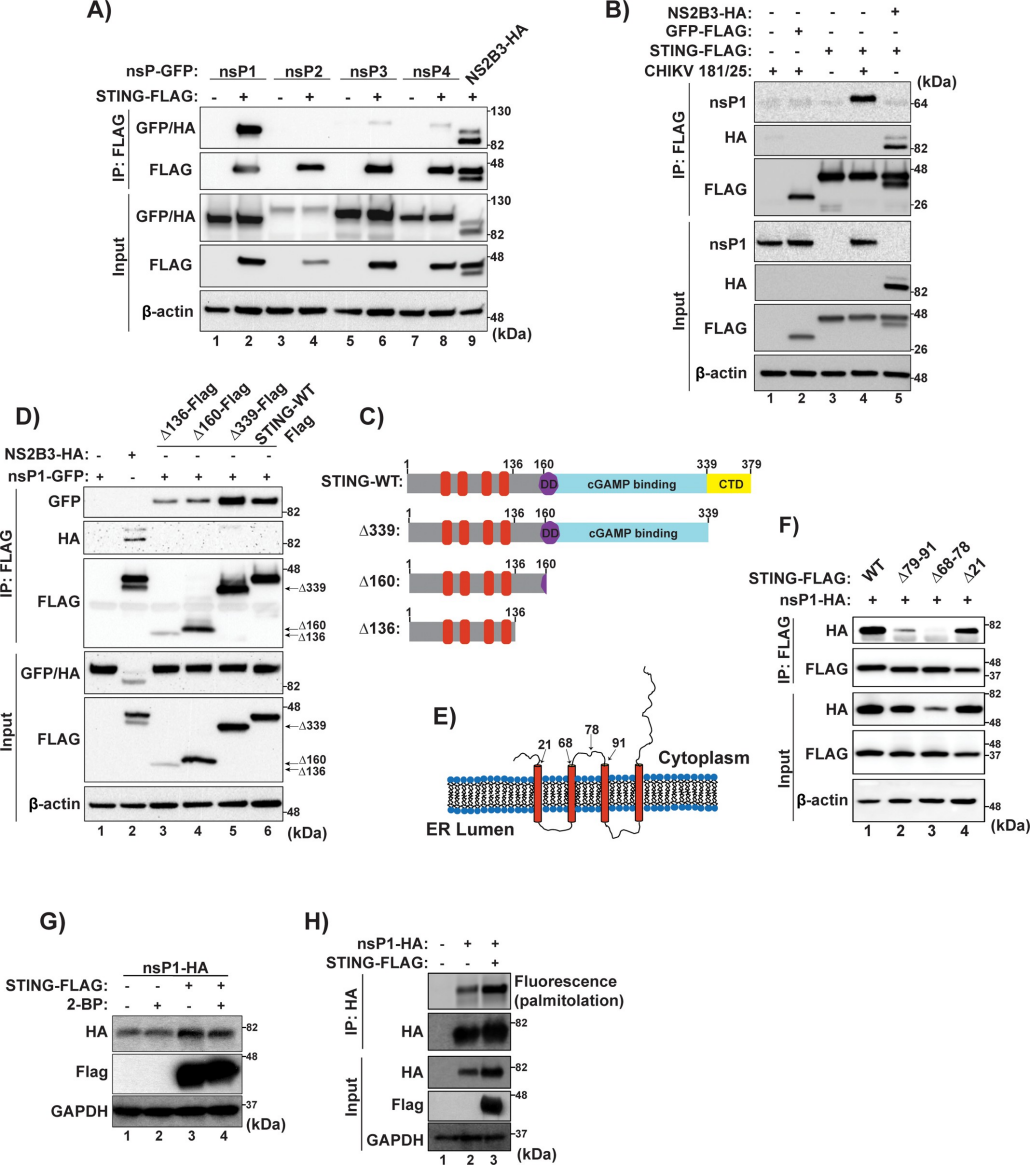
CHIKV nsP1 interacts with and is stabilized by STING. **(A)** Flag-tagged STING was co-expressed in HEK-293T cells, individually with nsPs 1–4 of CHIKV-RT. 24 hpt, cells were lysed and an immunoprecipitation preformed against the Flag-epitope. Protein interactions were visualized via SDS-PAGE followed by immunoblotting (data representative of two independent experiments). **(B)** HEK-293T cells were transfected with indicated constructs and allowed to rest for 16 hrs. After resting, cells were infected with either mock or CHIKV 181/25 (MOI = 5.0). 12 hpi cells were lysed and an immunoprecipitation preformed against a Flag epitope. Protein interaction was analyzed via SDS-PAGE followed by immunoblotting (data representative of two independent experiments). **(C)** Diagram of STING deletion constructs used for mapping nsP1-STING interactions. **(D)** HEK-293Ts were transfected with CHIKV-RT nsP1 and the different STING mutants indicated in (C) and cells were lysed 24 hrs post transfection. An immunoprecipitation was performed against the Flag epitope and protein samples were then analyzed via SDS-PAGE and immunoblotting performed as described previously (data representative of two independent experiments). **(E)** Schematic of STING inserted in the ER membrane highlighting regions deleted which are located in cytosolic facing domains (schematic representative of poor artistic skill). **(F)** 293T cells were transfected with internal deletion STING constructs and nsP1. Cells were lysed 24 hpt and an anti-flag IP was performed followed by SDS-PAGE and immunoblotting (data representative of three independent experiments). **(G)** HEK-293T cells were transfected cells with indicated constructs overnight followed by treatment with 100 uM 2-BP for 24 h. Cells were lysed and analyzed via Western blotting. **(H)** HEK-293T cells were transfected with indicated constructs overnight and treated for 1 h with 20 uM alk-16 palmitoylation chemical reporter reagent prior to cell lysis. Immunoprecipitation was performed against the HA epitope followed by click chemistry reaction with azido-rhodamine for visualization of protein palmitoylation via fluorescence gel scanning. (G & H) data representative of two independent experiments.

Interactions of CHIKV nsPs with STING were tested by immunoprecipitation against the Flag tag from total cell lysates and visualized via WB. A clear interaction of CHIKV nsP1 with STING was noted in an overexpression system (Fig 6A). Next, the nsP1 interaction was tested in the context of CHIKV 181/25 infection. Once again, the interaction of nsP1 with STING was observed, indicating that this interaction occurs in the context of viral infection (Fig 6B). To better characterize the interaction between nsP1 and STING, deletion mutants of STING were generated (Fig 6C). Sequential deletions of STING were made from the C-terminus of the protein to delete functional domains, but so as not to disrupt the insertion of the protein in the ER membrane or affect subcellular localization. STING Δ339 eliminates the C-terminal domain (CTD) of STING, which includes a critical interaction region for Tank Binding Kinase 1 interaction[64, 65]. The Δ160 mutant deletes the functional cyclic GMP-AMP (cGAMP) binding ability of the protein and the dimerization domain (DD), while the Δ136 mutant leaves only the transmembrane spanning portion of STING[65]. CHIKV nsP1 was then co-expressed with the deletion mutants of STING and an immunoprecipitation was performed (Fig 6D). Each mutant co-precipitated nsP1, indicating that interaction of nsP1 with STING only requires the transmembrane spanning domains of the innate immune signaling protein.

Given the predicted structure of STING as a four-pass transmembrane protein there are only two regions of STING exposed to the cytosol with which nsP1 could be interacting, the first 21 aa at the N-term or a cytosolic loop region spanning approximately amino acids 68–91[49, 65]. Deletion mutants of STING were generated lacking the first 20 aa at the N-term or which had small deletions in the cytosolic loop of the protein, either aa 68–78 or aa 79–91, respectively (Fig 6E). Immunofluorescence of HEK-293Ts transfected with STING internal deletion variants represented in 6E showed no significant changes in subcellular localization of the proteins (S3 Fig). Co-expression of nsP1 with a Δ20 mutant of STING illustrated that the N-Terminal tail of STING is not critical for interaction, but rather that the nsP1-STING interaction is mediated by the 23aa cytosolic loop of STING (Fig 6F). Interestingly, a loss of nsP1s interaction with STING resulted in decreased expression of the viral protein (Fig 6F, lane 3). Alternatively, co-expression of nsP1 with full length STING significantly and specifically increased nsP1 expression (S4 Fig).

Recently it has been demonstrated that murine STING is palmitoylated at cysteine residues 88 and 91 and that this modification is critical in activation of the antiviral response against DNA viruses[50]. These residues are conserved between human and murine STING and are in the cytosolic loop region which nsP1 of CHIKV is interacting with. Additionally, it has been demonstrated that nsP1 of CHIKV is palmitoylated and inhibition of this post-translational modification has myriad effects ranging from altering replication kinetics to pathogenesis in mice[16–19, 66]. Because of this association we hypothesized that nsP1s interaction with STING could result in increased levels of palmitoylated nsP1 and this could serve as a mechanism by which STING stabilize nsP1 expression. Interestingly, STING mediated nsP1 stabilization was independent of palmitoylation as demonstrated by transfecting nsP1 with STING both in the presence and absence of a global palmitoylation inhibitor, 2-Bromopalmitate (Fig 6G), although there was an increase in the levels of palmitoylated nsP1 when the protein was co-expressed with STING (Fig 6H). The described STING-nsP1 interaction, also resulted in a significant inhibition of the IFNβ promotor activation by cGAS-STING overexpression (S2B Fig). These data provide the first description of CHIKV interaction with STING. Furthermore, we mapped the interaction to a 23aa cytosolic loop of STING and demonstrates that this interaction could have a pro-viral role with respect to increasing the amount of nsP1 in infected cells in a STING dependent manner, while downregulating the IFNβ production pathway.

## Discussion

cGAS and STING have been previously implicated in restricting the replication of alphaviruses[21, 23, 25]. In MEF and RAW 264.7 cells deficient for cGAS or STING, we observed that the individual proteins in this DNA-sensing pathway serve to functionally restrict CHIKV replication and infectious particle release. These data are in accordance with previous publications which have implicated cGAS and STING as restriction factors for CHIKV replication[21, 23, 25] and provide further evidence that this pathway is important in inhibiting not only DNA viruses but also different class IV RNA viruses.

Similar to what our group has described during DENV infection[38], we also observed the production of distinct puncta of extranuclear DNA upon CHIKV infection. As defined sub-cellular compartmentalization of DNA is fundamental to the normal lifecycle of eukaryotic cells the presence of DNA in the cellular cytoplasm results in a strong type-I IFN response mainly mediated by cGAS-STING[67, 68]. The identity of the mis-localized DNA in CHIKV infected cells has yet to be understood, but its presence in the cytoplasm by definition serves as a danger associated molecular pattern (DAMP)[47, 69, 70]. When the cGAS-STING pathway was stimulated with either a DNA virus or *E*. *coli* DNA, we observed a replication dependent inhibition of *Ifnß* transcripts. Interestingly, there was a slight reduction in *Ifnß* transcripts when the cells were treated with a UV-inactivated CHIKV (UVC). This could be because UVC treatment resulted in the cells being refractory to infection with MVA; however, we did not observe a reduction in MVA replication measured by quantitative PCR. Another possibility was that a viral factor intrinsic to the nucleocapsid was responsible for the reduction in cGAS-STING dependent signaling observed. Indeed, exogenous expression of CHIKV capsid was sufficient to reduce cGAS expression and was able to significantly inhibit cGAS-STING mediated induction of a type-I IFN reporter. Immunoblotting of infected primary human fibroblasts revealed that endogenous cGAS is degraded as early as 4 hrs post infection and that this degradation is independent of global transcriptional or translational repression induced by CHIKV. The ability of a viral structural protein to reduce cGAS expression explains the rapid loss of cGAS protein levels at time-points prior to a full replication cycle of the virus with *de novo* production of viral proteins. Furthermore, capsid mediated degradation of cGAS explains the slight reduction in cGAS-STING signaling observed when cells were treated with UVC prior to stimulation with MVA or *E*. *coli* DNA.

Here, no data is presented identifying antagonism of cGAS-STING signaling at the level of STING, however, given the current view of cGAS dependent STING signaling, the degradation of cGAS by CHIKV, by definition, leads to an inhibition of cGAS-STING signaling. Further studies must be performed to understand if during CHIKV infection, hallmarks of STING activation are present including phosphorylation of serine 366, interactions with TBK1, or a subcellular re-localization of STING to the Trans-Golgi-Network (TGN). The role of autophagy during CHIKV infection is cell type specific and has been demonstrated to have both pro and antiviral effects[56, 59, 71, 72]. In addition to canonical antiviral signaling, other antiviral functions of STING have been documented including sensing of viral fusion[73, 74] and translational repression[40]. Research regarding the function of these alternative STING-dependent pathways during CHIKV infection is critical in developing a more complete understanding of the interplay of CHIKV with the cGAS-STING pathway.

Interestingly, nsPs 1–3 of CHIKV were also able to inhibit cGAS-STING mediated induction of a type-I IFN promotor, suggesting that multiple CHIKV proteins may work individually or in concert to antagonize cGAS-STING signaling during viral infection. Antagonism mediated by the non-structural proteins was not due to any direct effects on the levels of cGAS or STING expression indicating that they do not modulate the pathway via alteration of cGAS or STING protein levels. With these data, no direct mechanistic conclusions can currently be drawn as to inhibition of cGAS-STING signaling by nsPs 1–3 of CHIKV. They do however provide insight that degradation of cGAS may only be part of a repertoire of methods by which CHIKV antagonizes cytosolic DNA sensing.

We observed an interaction of nsP4 with cGAS, however, nsP4 did not significantly alter the activity of a type-I IFN reporter system. Interestingly, for Sindbis virus, another alphavirus, nsP4 has been shown to be degraded during infection via the proteasome by the N-end rule pathway during infection[75]. Thus, degradation of cGAS could be mediated partially by nsP4, but only when the host protein is degraded by the proteasome in the context of infection. This potential alternative protein degradation mechanism would explain why there is no total cGAS recovery during autophagy inhibition in CHIKV infection. Additionally, for other alphaviruses it has been demonstrated that nsP4 interacts with all three of the other non-structural proteins[76–78] and the viral RdRp, nsP4 is the first protein to be cleaved from the polyprotein nsP1-4 upon CHIKV infection. This early cleavage event in conjunction with replicative intermediates produced immediately upon infection could synergistically result in antagonism of cGAS dependent signaling, but only in the context of infection.

By using two complementary methods to inhibit autophagic flux, a chemical inhibitor, 3-MA, and siRNA knockdown of ATG7, a critical factor in initiation of autophagosomes, we observed a significant recovery of cGAS expression in CHIKV infected cells. Specifically, when cGAS levels were normalized to their respective non-targeting controls and ATG7 expression was taken into account, there was an 8.1 fold increase in cGAS expression in CHIKV-infected conditions versus a 2.6 fold increase in mock-infected cells. Importantly, a reduction in the amount of LC3II was observed in knockdowns of ATG7 when compared to the non-targeting controls, indicating the knockdowns were functionally reducing autophagy. Interestingly, it has been reported that there is an ATG5/ATG7 independent form of autophagy in murine embryonic fibroblasts (MEFs)[79]. This ATG5/ATG7 independent macro-autophagy did not result in the accumulation of LC3II in cells. Because LC3II was observed in the ATG7 knockdowns, ATG7 dependent autophagy was reduced but we cannot rule out whether or not ATG7 independent autophagy, plays a role in cGAS degradation during CHIKV infection. Furthermore, it has been demonstrated that during infection, CHIKV capsid interacts with selective autophagy mediator p62 and this interaction results in capsid degradation via autophagy[71]. Another study demonstrated that p62 can interact with and modulate cGAS expression via autophagic degradation[80]. It is thus possible that the degradation of cGAS by CHIKV capsid is mediated via an intermediary interaction between cGAS-p62- and -capsid which results in both being degraded during infection.

CHIKV nsP1 was found to interact with STING. nsP1 is the only non-structural protein of CHIKV which is anchored to cellular membranes[81–83] and STING is a transmembrane protein which has been shown to localize to the endoplasmic reticulum (ER), mitochondrial associated membranes (MAM), and cytoplasmic vesicles[42, 84]. It is possible that the interaction between nsP1 and STING is enhanced because of their proximity due to membrane association. The interaction between nsP1 and STING was mapped to a 23 amino acid cytosolic loop region of the protein. It is possible that through this interaction, nsP1 is disrupting dimerization of STING and thus dampening antiviral responses. However, the cytosolic loop domain of STING has not been shown to function in dimerization, so that possibility is unlikely. Further work must be done to understand the residues required for STING interaction from the molecular perspective of nsP1 as well as if there are differences between human and murine STING with respect to the nsP1 interaction as previously reported for DENV NS2B3[39].

It has been reported that STING interacts with both RIG-I and MAVS and that STING null cells have reduced type-I IFN production when stimulated with vesicular stomatitis virus (VSV) or Sendai virus (SeV)[41, 42, 85]. The nsP1-STING interaction, could thus be affecting RIG-I-like receptor (RLR) signaling by disrupting the innate immune signaling complex of RIG-I-MAVS-STING, resulting in an inhibition of RLR mediated type-I IFN induction during CHIKV infection. Alternatively, this interaction could serve to inhibit another reported antiviral role of STING: translational repression during RNA virus infection[40]. Furthermore, no research has directly addressed whether or not CHIKV is able to inhibit RLR mediated antiviral sensing or signaling. For a complete understanding of CHIKV mediated antagonism of innate immunity, it will be critical to study potential viral antagonism of RLR sensing as well as the role of STING’s crosstalk with these sensors.

Interestingly, the nsP1-STING interaction serves to increase protein levels of nsP1 in cells specifically when compared to a GFP control. We found that the increase in nsP1 levels resulted in increased palmitoylated nsP1 in cells, although an inhibitor of palmitoylation, 2-BP, did not alter the increased nsP1 expression indicating that the process of palmitoylation was not required for the increase in nsP1 expression. Palmitoylated nsP1 has been previously shown to enhance replication kinetics and pathogenesis of alphaviruses in mice[18, 19] while mutant CHIKV viruses with non-palmitoylatable nsP1s showed severely altered replication kinetics and nsP1 membrane association was disrupted[16]. This provides a potential dual role for the nsP1-STING interaction: simultaneously inhibiting STING mediated antiviral signaling while being stabilized, thus increasing the total levels of nsP1. Furthermore, CHIKV nsP1 has been identified as a druggable target[86]. Currently, there are no complete protein models of either STING or CHIKV nsP1, hindering *in silico* modeling of protein-protein interactions. By determining the cytosolic loop domain of STING as being critical for the nsP1-STING interaction and identifying that STING increases nsP1 expression, our data provide valuable information for understanding the molecular basis of interfacing between these two proteins which can aid in small molecule inhibitor screening and design.

Significant evidence supports that the DNA sensing pathway of cGAS and STING plays an important role in restricting RNA virus infections and viral antagonists of cGAS and STING have been identified for both flaviviruses and human coronaviruses[38, 39, 42, 44, 45, 87, 88]. In this work we sought to understand if CHIKV interacted with or restricted the cGAS-STING innate immune signaling pathway. Taken together, these data have strong potential implications for the rational design of attenuated CHIKV viruses, provide information regarding nsP1 for small molecule inhibitor design, and identify, for the first time, direct antagonism of a cytosolic DNA sensing pathway by CHIKV.

## Materials and methods

### Cell lines

Human foreskin fibroblast (HFF-1) cells were obtained through ATCC (ATCC SCRC-1041) and cultured in Dulbecco’s Modified Essential Medium (DMEM) supplemented with 10% fetal bovine serum (FBS). Human embryonic kidney-293T (HEK-293T) cells were also obtained from ATCC (ATCC CRL-3216) and were grown in DMEM supplemented with 5% FBS, 100 U ml^-1^ L-glutamine, 100 U ml^-1^ penicillin/streptomycin. Mosquito cells from *Aedes albopictus* mosquitos, clone C6/36 (obtained originally from J. Munoz-Jordan, CDC, Puerto-Rico) were maintained in RPMI medium with 10% FBS at 33°C. Baby hamster kidney cells (BHK) were passaged in Minimum Essential Medium (MEM) Alpha + GlutaMAX-l purchased from Gibco and supplemented with 10% FBS, 100 U ml^-1^ penicillin/streptomycin, and 10 mM 4-(2-hydroxyethyl)-1-piperazineethanesulfonic acid (HEPES). U2OS cells were a gift from Dr. Carolyn Coyne’s laboratory and were maintained in media used for the HEK-293Ts. HEK-293T-IFNß reporter cells, previously described[89] were grown as stated for HEK-293Ts. Vero cells were purchased from ATCC (ATCC CCL-81) and were maintained in media described for HEK-293Ts. All tissue culture reagents were purchased from Invitrogen. MEFs, WT and GT, were a gift from Dr. Jonathan Miner (WUSTL) and were maintained in (DMEM) supplemented with 20% fetal bovine serum (FBS). RAW 264.7 cells were purchased from Invitrogen and cultured according to the manufacturer’s instructions. Catalog numbers: WT: rawl-isg, cGAS KO: rawl-kocgas, and STING KO: rawl-kostg.

### Viruses

The chikungunya 181/25 (CHIKV 181/25) strain, originally derived from a patient isolate in Thailand[90], used in this study was kindly provided by Dr. St. Patrick Reid at the University of Nebraska, Omaha. The virus was passaged one time in Vero cells and supernatants were collected, clarified, and stored at -80°C. Viral titers were determined by limiting dilution plaque assay on BHK-21s as previously described for dengue virus (DENV)[91]. Newcastle Disease (NDVB1-GFP), originally obtained from Dr. Adolfo Garcia-Sastre, were grown in nine day old embryonated chicken eggs and tittered via TCID_50_ on chicken embryonic fibroblasts (CEFs) as previously described[92, 93]. Vaccinia virus aliquots were a kind gift from Dr. Nacho Mena, of the Gracia-Sastre laboratory. Dengue virus serotype 2 (DENV-2) strain 16681 was grown for six days as previously described in C6/36 cells[94]. CHIKV Indian Ocean Lineage (CHIKV IOL) was generated from the La Reunion (strain 06–049) infectious clone (Coffey et al, 2011). To generate infectious virus, the infectious clone plasmids were linearized overnight with NotI, purified, and used for *in vitro* transcription with the SP6 mMachine kit (Ambion). *In vitro* transcribed RNA was phenol:Chloroform extracted, ethanol precipitated, aliquoted at 1 μg/μl and stored at -80°C. 10 μg of *in vitro* transcribed RNA was electroporated into BHK cells and virus was harvested at 48 hours post electroporation. Working virus stocks were generated by passaging the virus in BHK cells for 24 hours. Viral titers were determined by plaque assay on Vero cells.

### UV-inactivation of virus

UV-C bulb was placed 3 inches from the 1 ml viral aliquots in 12-well culture dishes (Corning) with the lid removed. Plates were placed on a magnetic stir plate and sterile magnetic stir bars were placed in each well. UV-inactivation was allowed to proceed for 10 min at room temperature. Viral aliquots were then stored at 4°C for future use. UV-C bulb used: G8T5. 8W. The wavelength of light was 254 nm.

### Interferon-ß reporter assay

The ability of different CHIKV nsPs to inhibit the induction of the IFNß reporter was assessed in HEK-293T cells stably expressing firefly luciferase under the control of an IFN-ß promotor (293T-IFNß), previously described[89]. 50,000 293T-IFNß cells were transiently reverse transfected using Lipofectamine 2000 (Thermo) with 100 ng total DNA per well of different constructs expressing either human cGAS-pCMV6 (Origene), human STING-(pcDNA), DENV NS2B3-(pCAGGS), CHIKV GFP-nsPs plasmids, (kindly provided by Dr. Subhash G Vasudevan (Duke-NUS Graduate Medical School, Singapore), CHIKV Capsid (pTR-600) or pCAGGS with no coding insert (empty vector (EV)), in 96-well plates, using Lipofectamine 2000 reagent (Invitrogen) per the manufacturer’s protocol. 40 hours post transfection, IFN-ß promotor induction was measured using the neolite luminescence reporter gene assay system (PerkinElmer) per manufacturer’s protocol. Western blots were performed as described in “Immunoblot analysis” section.

### Infection of HFF-1s

Primary human foreskin fibroblasts (HFF-1) were seeded in 12-well plates at a density of 1.8X10^5^ cells/well. 24 hours after seeding, cells were treated with either mock (DMEM), NDVB1-GFP at an MOI of 0.1, or CHIKV, either CHIKV IOL or 181/25 at an MOI of 0.1. Infections were allowed to proceed for 1hr in a total volume of 100 μl of sera-free DMEM. After 1 hr, infection media was removed and cells were re-fed with 1 ml of HFF-1 media (DMEM with 15% FBS). 4, 12, 24, and 48 hours post infection supernatants were collected and quick-frozen in dry ice/ethanol then stored at -80°C. At the selected time-points, RNA from cells was collected according to the manufacturers’ protocol using the Quick-RNA Mini-Prep (Zymogen) and stored at -80°C. Protein lysates were collected by re-suspending cells in RIPA lysis buffer (Sigma Aldrich) and were subsequently stored at -80°C.

### Co-treatment of HFF-1s

Prior to infection, 2.0X10^5^ HFF-1s were seeded in 12-well culture dishes (Corning). Cells were then treated with mock, CHIKV 181/25 or UV-inactivated CHIKV 181/25 (UVC) at an MOI of 5.0. After primary treatment, cells were allowed to rest for 6hrs. The secondary treatments were administered 6hrs post primary treatment. Cells were infected with either mock or modified vaccinia Ankara (MVA) at an MOI of 2.0 to induce the RLR or cGAS-STING pathways and RNA was collected 6 and 12 hrs post-secondary treatment. Alternatively, cGAS-STING induction was performed via mock or *E*. *coli* DNA (1ug/well) (InvivoGen) transfection using Lipofectamine 2000 according to manufacturer’s instructions. RNA was collected at 6 and 12 hrs post transfection. All RNA collections were performed according to the manufacturer’s protocol using the Quick-RNA Mini-Prep (Zymogen). Determination of induction of innate immune signaling gene transcripts was determined by normalizing all conditions to *rps11* and then determining fold induction over respective mock E.G. induction of *Isg15* transcripts as a result of MVA infection was determined by comparing UVC ➔ MVA over UVC➔mock (primary ➔ secondary) conditions.

### RNA isolation and reverse transcription (RT)

RNA from cells was extracted using Quick-RNA Mini-Prep (Zymogen) according to the manufacturers protocol (including the in-column DNase treatment). Concentration of ribonucleic acid was determined via spectrophotometer at 260nm. RNA was then stored at -80°C until RT reaction. RT reaction was done with the iScript cDNA synthesis kit (Bio-Rad) utilizing random hexamer priming, according to the manufacturer’s instructions with 500–1000 ng of total RNA.

### RT-qPCR

RT-qPCR was used to quantify relative levels of gene expression in infected and uninfected cells and was performed using the iQ SYBR green Supermix (BioRad) according to the manufacturer’s instructions. The BioRad 1000C Thermal Cycler was used with the following PCR profile: 94°C for 3min followed by 40 cycles of 94°C for 15s then 60°C for 30s. Quantification of gene expression was performed based on Ct values of a given gene normalized to the housekeeping gene, *Rps11*, *Gapdh*, or both where indicated.

### Immunoblot analysis

Cellular lysates were obtained by incubating cells in RIPA lysis buffer (Sigma Aldrich) supplemented with EDTA-free, cOmplete ULTRA Tablets, mini (Roche) for 30 min on ice. Quantification of protein in cellular lysates was performed via colorimetric Bradford Assay (Bio-Rad) utilizing bovine serum albumin (BSA) for generation of a standard curve. Cellular lysates were re-suspended, in a 1:1 ratio, in 2X Laemmli sample buffer (Bio-Rad) supplemented with 2-mercaptoethanol and boiled at 100°C for 10min in a heating block (Fisher Scientific). All samples were then loaded on polyacrylamide-SDS gels and the denatured proteins were separated by electrophoresis via conventional methods. Protein was then transferred to nitrocellulose membranes (Bio-Rad). Blots were blocked with phosphate buffered saline (PBS) with 5% milk for one hour at room temperature. Antibodies used: cGAS (D1D3G), STING (D2P2F), RIG-I (D33H10), ATG7 (D12B11) (Cell Signaling Technology) at a 1:1000 dilution, anti-FLAG (F7425), anti- ß-actin (A2228), and anti haemagglutinin (HA) (H3663) (Sigma Aldrich) at a 1:5000 dilution, anti-GFP (MA5-15256) (Invitrogen), anti-Chikungunya virus clone 6A11 (MABF2051), anti-puromycin clone 12D10 (MABE343) (EMD Millipore). Antibodies against CHIKV 181/25 were kind gifts from Drs. Stapleford and Reid. Secondary antibodies against mouse (NA931V) and rabbit (NA934V) (GE Healthcare). Detection of immunocomplexes were performed using SuperSignal chemilumisescence system (Thermo). Densitometry analysis was performed using ImageJ software.

### Immunofluorescence

293T cells were seeded on 12-well glass-bottomed plates (MaTtek) which had been pre-coated with 0.1% polylysine for 1hr at RT or HFF-1s were seeded directly on the glass-bottomed plates without pretreatment. 24hrs post transfection or infection, cells were fixed at RT with 4.0% formaldehyde then permeabilized with 0.1% Triton-X before blocking with 4% BSA in PBS for 1hr at RT. Cells were incubated overnight at 4°C with primary antibodies: (H3663: Sigma Aldrich), anti-ssDNA (MAB3034: EMD Millipore), anti-nsP2 (provided by Dr. Stapleford), anti-Flag (F7425: Sigma), or anti-calnexin (MA3-027: Invitrogen). Cells were then incubated with for 1hr at RT with Alexa fluor-conjugated anti-mouse 488, anti-rabbit 633 (Life Technologies), 1 μg ml^–1^ DAPI (Invitrogen) or phalloidin (A30107: Invitrogen) as indicated. Confocal imaging was performed using a Zeiss LSM 880 with Airyscan. Images were collected at 16 bits and a resolution of 1024 X 1024 pixels. 3D images were created via reconstruction of Z-Stacks in Zen Blue software.

### Generation of STING constructs

Deletions of functional regions of STING were generated and cloned into the pTR-600 mammalian expression vector using EcoRI and BamHI restriction sites utilizing the In-Fusion HD Cloning Kit (Clontech).

### Immunoprecipitation assay

Immunoprecipitation was performed using the EZview Red ANTI-FLAG M2 Affinity Gel (Sigma Aldrich). Briefly, affinity gel was washed 3X in lysis buffer (RIPA lysis buffer (Thermo) supplemented with cOmplete mini EDTA-free protease inhibitor (Roche). Whole cell lysates were mixed with the affinity gel and incubated for 1hr at 4°C, rotating. After incubation, affinity gel was washed 4X for 10 min each in lysis buffer. Following washes, the affinity gel was re-suspended in 50 μl 2X Laemmli buffer (Bio-Rad) with 2-mercaptoethanol (Sigma Aldrich) and boiled for 10 min at 100°C. Samples were then stored at -20°C until immunoblot analysis.

### Chemical inhibition of autophagy

1.0X10^6^ HEK-293Ts were reverse transfected with 200 ng of human cGAS-pCMV6 (Origene), using Lipofectamine 2000 (Thermo) according to the manufacturer’s protocol. 16 hrs post transfection, cells were infected with CHIKV 181/25 at an MOI of 10.0 in serum free DMEM for 1 hr at 37°C. After 1 hr, cells were re-fed with DMEM supplemented with 5% FBS, 100 U ml^-1^ L-glutamine, 100 U ml^-1^ penicillin/streptomycin. 10 hrs post infection, cells were treated with 3-MA (5 mM, Sigma), diluted in UltraPure distilled water (Invitrogen). 10 hrs post drug treatment, cellular lysates were obtained by incubating cells in RIPA lysis buffer (Sigma Aldrich) supplemented with EDTA-free, cOmplete ULTRA Tablets, mini (Roche) for 30 min on ice. Lysates were stored at -80°C until immunoblot analysis.

### siRNA transfection and CHIKV Infection

7.5 x 10^4^ U2OS cells were seeded in 12-well plates and transfected with 10 nM of negative control siRNA (Silencer Select Negative Control #1 siRNA (Cat #: 4390843)) or ATG7 siRNA (5’-GCACUAGAGUGUGCAUAUGTT-3’. Forward transfection of siRNA was performed 24 hours after cells were seeded using Lipofectamine 2000 reagent according to the manufacturer’s protocol. 1 hour prior to transfection, media was removed and 1 mL of 2% FBS DMEM was added. siRNA and lipofectamine were diluted in OPTI-MEM to achieve appropriate working concentrations. Media was changed 4 hours post transfection with 10% FBS DMEM. Mock and CHIKV 181/25 infections were performed 48 hrs post siRNA transfection. Cells were infected with CHIKV 181/25 at a MOI 10 in 150 μL of DMEM and incubated at 37°C for 1 hour, rocking the plate every 10 minutes. Mock infection was performed with 150 μL of DMEM. 1-hour post infection, virus was removed and fresh 293T media was added. Cell were lysed with RIPA and collected for processing 24 hours post infection.

### Palmitoylation assay

Studies of protein palmitoylation were performed according to detailed published protocols[95–97]. In brief, transfected cells were treated for 1 h with the alk-16 (20 uM) chemical reporter of protein palmitoylation. Proteins of interest were immunoprecipitated from cell lysates and reacted via the copper(I)-catalized azide alkyne cycloaddition reaction (“click chemistry”) with azido-rhodamine (kindly provided by Dr. Howard Hang of the Rockefeller University) for visualization of protein acylation via fluorescence gel scanning on a Typhoon 9400 (Amersham) fluorescence imager. Western blotting of the samples provided controls for loading and sample inputs. For inhibition of protein palmitoylation, transfected cells were treated for 24 h with 100 uM 2-bromopalmitate (Sigma).

### Statistical analysis

Unpaired, two-tailed, Student’s t-test was used for direct comparisons while one way or two way ANOVAs with Tukey’s multiple comparisons we used for viral growth curves or multiple comparisons. Specific analysis used for respective figures are listed in the figure legends. P-values were determined to be significant when p < 0.05. Relevant p-value cutoffs used are listed in figure legends. No samples were excluded when analyzing these data.

## Supporting information

S1 Fig Replication kinetics and innate immune response profile of CHIKV IOL and CHIKV 181/25. Human foreskin fibroblasts (HFF-1) were either mock infected or infected with CHIKV IOL, CHIKV 181/25, or NDVB1 at an MOI of 0.1. Supernatants and RNA were collected 4, 12, 24, and 48 hpi. **(A)** Plaque assay on BHKs of supernatants from infected HFF-1s. **(B)** RT-qPCR analysis of infected cells measuring *nsP2*. **(C-F)** RT-qPCR analysis of innate immune transcripts in infected cells at 4 and 24 hpi with primers specific for *Ifnß*, *Isg15*, *Tnf-a*, and *Il-1ß*. Data shown representative of two independent experiments. All RT-qPCR genes represented in this figure were normalized to *rps11* then represented as vRNA/rps11 or fold over mock as indicated. Data are represented as means ± SD (n = 3). Statistical analysis was done by student’s t test (A & B) or two way ANOVA with Tukey’s multiple comparisons (C-F) (ns = not significant, * = p<0.05, ** = p<0.01, *** = p<0.001, **** = p<0.0001).
(TIF)

S2 Fig CHIKV non-structural protein modulation of cGAS and STING. **(A)** Human liver cells (Huh7) which do not endogenously express cGAS or STING, human foreskin fibroblasts (HFF-1), human embryonic kidney (HEK-293T), and HEK-293T cells stably expressing an IFNβ promoter driving the production of firefly luciferase (293T-IFNβ-FFluc) were lysed and protein was analyzed via SDS-PAGE. Expression levels of endogenous cGAS, STING, and RIG-I are shown for the respective cell types. Data representative of two independent experiments. **(B)** 293T-IFNb-FFluc cells were transfected with cGAS and STING in conjunction with empty vector (vector), or the indicated viral proteins (nsPs 1–4 of CHIKV-RT). Cells were allowed to rest for 36hrs before lysis for collection of protein or quantification of luminescence. **(C)** Input protein expression for reporter experiment (B) was visualized via SDS-PAGE followed by immunoblotting. Data representative of four independent experiments. Data are represented by means ± SD (n = 3), fold induction over mock. Statistical analysis was done with student’s t tests (* = p<0.05, ** = p<0.01, *** = p<0.001). **(D)** HEK-293T cells were transfected with indicated constructs and cells were lysed 24 hpt. DENV-2 NS2B3 served as a positive control for STING cleavage/degradation while the catalytically inactive NS2B3 S135A was used as a negative control. GFP tagged CHIKV-RT nsP constructs were used to test for degradation or cleavage of STING. Protein lysates were analyzed via SDS-PAGE and subsequent immunoblotting. Data representative of one independent experiment. **(E)** HEK-293T cells were transfected with indicated constructs (nsPs 1-4-HA CHIKV-RT) and cells were allowed to rest for 24 hrs before lysis. Lysates were subjected to immunoprecipitation against a Flag epitope and proteins were visualized via SDS-PAGE and immunoblotting. Data representative of three independent experiments. **(F)** Indicated constructs were expressed in 293T cells and cells were allowed to rest for 16 hrs. After resting, cells were infected with either mock or CHIKV 181/25 (MOI = 5.0). 12 hpi cells were lysed and an immunoprecipitation preformed against a Flag epitope. Protein interaction was analyzed via SDS-PAGE followed by immunoblotting. Data representative of two independent experiments. **(G)** Transfected HEK-293T cells were fixed and permeabilized 24 hrs post transfection then stained for indicated proteins for imaging at. Imaging performed with a Zeiss LSM 880 with Airyscan. Orthogonal views of intersecting red lines are displayed top and right of the respective images. Scale bars = 20um. Data are representative of two independent experiments.
(TIF)

S3 Fig Subcellular localization of STING internal deletion constructs. HEK 293Ts were transfected with empty vector or indicated STING constructs. 24 hpi cells were fixed with 4% formaldehyde and permeabilized with 0.01% triton-X before staining with antibodies specific for Flag-(STING: purple), Calnexin (aqua), Phalloiden (grey), or DAPI (blue). Cells were visualized using a Zeiss LSM 880 with Airyscan. Scale bars = 10um. Data are representative of two independent experiments.
(TIF)

S4 Fig STING expression specifically stabilizes nsP1. HEK-293T cells were transfected with increasing concentrations of the indicated constructs and cells were lysed 24 hpt. Lysates were analyzed via SDS-PAGE and immunoblotting. Densitometry measurement of bands in figure blot performed with ImageJ software. Data are representative of two independent experiments.
(TIF)

## References

[1] Kuhn R. Togaviridae: the viruses and their replication. Fields virology. 2007;1:1001–22.

[2] Vega-Rua A, Zouache K, Girod R, Failloux AB, Lourenco-de-Oliveira R. High level of vector competence of Aedes aegypti and Aedes albopictus from ten American countries as a crucial factor in the spread of Chikungunya virus. J Virol. 2014;88(11):6294–306. Epub 2014/03/29. 10.1128/JVI.00370-14 24672026 PMC4093877

[3] Thiberville SD, Moyen N, Dupuis-Maguiraga L, Nougairede A, Gould EA, Roques P, et al Chikungunya fever: epidemiology, clinical syndrome, pathogenesis and therapy. Antiviral Res. 2013;99(3):345–70. Epub 2013/07/03. 10.1016/j.antiviral.2013.06.009 . 23811281 PMC7114207

[4] Borgherini G, Poubeau P, Jossaume A, Gouix A, Cotte L, Michault A, et al Persistent arthralgia associated with chikungunya virus: a study of 88 adult patients on reunion island. Clin Infect Dis. 2008;47(4):469–75. Epub 2008/07/10. 10.1086/590003 . 18611153

[5] Bouquillard E, Combe B. A report of 21 cases of rheumatoid arthritis following Chikungunya fever. A mean follow-up of two years. Joint Bone Spine. 2009;76(6):654–7. Epub 2009/12/01. 10.1016/j.jbspin.2009.08.005 . 19945329

[6] Burt F, Chen W, Mahalingam S. Chikungunya virus and arthritic disease. Lancet Infect Dis. 2014;14(9):789–90. Epub 2014/08/29. 10.1016/S1473-3099(14)70869-2 . 25164188

[7] Lumsden WH. An epidemic of virus disease in Southern Province, Tanganyika Territory, in 1952–53. II. General description and epidemiology. Trans R Soc Trop Med Hyg. 1955;49(1):33–57. Epub 1955/01/01. 10.1016/0035-9203(55)90081-x . 14373835

[8] Morrison TE. Reemergence of chikungunya virus. J Virol. 2014;88(20):11644–7. Epub 2014/08/01. 10.1128/JVI.01432-14 25078691 PMC4178719

[9] Robinson MC. An epidemic of virus disease in Southern Province, Tanganyika Territory, in 1952–53. I. Clinical features. Trans R Soc Trop Med Hyg. 1955;49(1):28–32. Epub 1955/01/01. 10.1016/0035-9203(55)90080-8 . 14373834

[10] Renault P, Solet JL, Sissoko D, Balleydier E, Larrieu S, Filleul L, et al A major epidemic of chikungunya virus infection on Reunion Island, France, 2005–2006. Am J Trop Med Hyg. 2007;77(4):727–31. Epub 2007/11/06. . 17978079

[11] Wahid B, Ali A, Rafique S, Idrees M. Global expansion of chikungunya virus: mapping the 64-year history. Int J Infect Dis. 2017;58:69–76. Epub 2017/03/16. 10.1016/j.ijid.2017.03.006 . 28288924

[12] Arankalle VA, Shrivastava S, Cherian S, Gunjikar RS, Walimbe AM, Jadhav SM, et al Genetic divergence of Chikungunya viruses in India (1963–2006) with special reference to the 2005–2006 explosive epidemic. J Gen Virol. 2007;88(Pt 7):1967–76. Epub 2007/06/08. 10.1099/vir.0.82714-0 . 17554030

[13] Rupp JC, Sokoloski KJ, Gebhart NN, Hardy RW. Alphavirus RNA synthesis and non-structural protein functions. J Gen Virol. 2015;96(9):2483–500. Epub 2015/07/30. 10.1099/jgv.0.000249 26219641 PMC4635493

[14] Strauss EG, Rice CM, Strauss JH. Sequence coding for the alphavirus nonstructural proteins is interrupted by an opal termination codon. Proc Natl Acad Sci U S A. 1983;80(17):5271–5. Epub 1983/09/01. 10.1073/pnas.80.17.5271 6577423 PMC384235

[15] Li G, Rice CM. The signal for translational readthrough of a UGA codon in Sindbis virus RNA involves a single cytidine residue immediately downstream of the termination codon. J Virol. 1993;67(8):5062–7. Epub 1993/08/01. 10.1128/JVI.67.8.5062-5067.1993 8331741 PMC237898

[16] Zhang N, Zhao H, Zhang L. Fatty acid synthase promotes the palmitoylation of Chikungunya virus nsP1. J Virol. 2018 Epub 2018/11/09. 10.1128/JVI.01747-18 . 30404808 PMC6340048

[17] Spuul P, Salonen A, Merits A, Jokitalo E, Kaariainen L, Ahola T. Role of the amphipathic peptide of Semliki forest virus replicase protein nsP1 in membrane association and virus replication. J Virol. 2007;81(2):872–83. Epub 2006/11/10. 10.1128/JVI.01785-06 17093195 PMC1797454

[18] Ahola T, Kujala P, Tuittila M, Blom T, Laakkonen P, Hinkkanen A, et al Effects of palmitoylation of replicase protein nsP1 on alphavirus infection. J Virol. 2000;74(15):6725–33. Epub 2000/07/11. 10.1128/jvi.74.15.6725-6733.2000 10888610 PMC112188

[19] Zusinaite E, Tints K, Kiiver K, Spuul P, Karo-Astover L, Merits A, et al Mutations at the palmitoylation site of non-structural protein nsP1 of Semliki Forest virus attenuate virus replication and cause accumulation of compensatory mutations. J Gen Virol. 2007;88(Pt 7):1977–85. Epub 2007/06/08. 10.1099/vir.0.82865-0 17554031 PMC2271122

[20] Solignat M, Gay B, Higgs S, Briant L, Devaux C. Replication cycle of chikungunya: a re-emerging arbovirus. Virology. 2009;393(2):183–97. Epub 2009/09/08. 10.1016/j.virol.2009.07.024 19732931 PMC2915564

[21] Gall B, Pryke K, Abraham J, Mizuno N, Botto S, Sali TM, et al Emerging Alphaviruses Are Sensitive to Cellular States Induced by a Novel Small-Molecule Agonist of the STING Pathway. J Virol. 2018;92(6). Epub 2017/12/22. 10.1128/JVI.01913-17 29263267 PMC5827377

[22] Olagnier D, Scholte FE, Chiang C, Albulescu IC, Nichols C, He Z, et al Inhibition of dengue and chikungunya virus infections by RIG-I-mediated type I interferon-independent stimulation of the innate antiviral response. J Virol. 2014;88(8):4180–94. Epub 2014/01/31. 10.1128/JVI.03114-13 24478443 PMC3993760

[23] Sali TM, Pryke KM, Abraham J, Liu A, Archer I, Broeckel R, et al Characterization of a Novel Human-Specific STING Agonist that Elicits Antiviral Activity Against Emerging Alphaviruses. PLoS Pathog. 2015;11(12):e1005324 Epub 2015/12/10. 10.1371/journal.ppat.1005324 26646986 PMC4672893

[24] Schilte C, Couderc T, Chretien F, Sourisseau M, Gangneux N, Guivel-Benhassine F, et al Type I IFN controls chikungunya virus via its action on nonhematopoietic cells. J Exp Med. 2010;207(2):429–42. Epub 2010/02/04. 10.1084/jem.20090851 20123960 PMC2822618

[25] Schoggins JW, MacDuff DA, Imanaka N, Gainey MD, Shrestha B, Eitson JL, et al Pan-viral specificity of IFN-induced genes reveals new roles for cGAS in innate immunity. Nature. 2014;505(7485):691–5. Epub 2013/11/29. 10.1038/nature12862 24284630 PMC4077721

[26] White LK, Sali T, Alvarado D, Gatti E, Pierre P, Streblow D, et al Chikungunya virus induces IPS-1-dependent innate immune activation and protein kinase R-independent translational shutoff. J Virol. 2011;85(1):606–20. Epub 2010/10/22. 10.1128/JVI.00767-10 20962078 PMC3014158

[27] Goubau D, Deddouche S, Reis e Sousa C. Cytosolic sensing of viruses. Immunity. 2013;38(5):855–69. Epub 2013/05/28. 10.1016/j.immuni.2013.05.007 . 23706667 PMC7111113

[28] Jensen S, Thomsen AR. Sensing of RNA viruses: a review of innate immune receptors involved in recognizing RNA virus invasion. J Virol. 2012;86(6):2900–10. Epub 2012/01/20. 10.1128/JVI.05738-11 22258243 PMC3302314

[29] McNab F, Mayer-Barber K, Sher A, Wack A, O'Garra A. Type I interferons in infectious disease. Nature Reviews Immunology. 2015;15:87 10.1038/nri3787 25614319 PMC7162685

[30] Schneider WM, Chevillotte MD, Rice CM. Interferon-stimulated genes: a complex web of host defenses. Annu Rev Immunol. 2014;32:513–45. Epub 2014/02/22. 10.1146/annurev-immunol-032713-120231 24555472 PMC4313732

[31] Fros JJ, Liu WJ, Prow NA, Geertsema C, Ligtenberg M, Vanlandingham DL, et al Chikungunya virus nonstructural protein 2 inhibits type I/II interferon-stimulated JAK-STAT signaling. J Virol. 2010;84(20):10877–87. Epub 2010/08/06. 10.1128/JVI.00949-10 20686047 PMC2950581

[32] Fros JJ, Major LD, Scholte FEM, Gardner J, van Hemert MJ, Suhrbier A, et al Chikungunya virus non-structural protein 2-mediated host shut-off disables the unfolded protein response. J Gen Virol. 2015;96(Pt 3):580–9. Epub 2014/11/15. 10.1099/vir.0.071845-0 . 25395592

[33] Fros JJ, van der Maten E, Vlak JM, Pijlman GP. The C-terminal domain of chikungunya virus nsP2 independently governs viral RNA replication, cytopathicity, and inhibition of interferon signaling. J Virol. 2013;87(18):10394–400. Epub 2013/07/19. 10.1128/JVI.00884-13 23864632 PMC3753987

[34] Akhrymuk I, Kulemzin SV, Frolova EI. Evasion of the innate immune response: the Old World alphavirus nsP2 protein induces rapid degradation of Rpb1, a catalytic subunit of RNA polymerase II. J Virol. 2012;86(13):7180–91. Epub 2012/04/20. 10.1128/JVI.00541-12 22514352 PMC3416352

[35] Meshram CD, Lukash T, Phillips AT, Akhrymuk I, Frolova EI, Frolov I. Lack of nsP2-specific nuclear functions attenuates chikungunya virus replication both in vitro and in vivo. Virology. 2019;534:14–24. Epub 2019/06/05. 10.1016/j.virol.2019.05.016 31163352 PMC7204530

[36] Maringer K, Fernandez-Sesma, A. Message in a bottle: lessons learned from antagonism of STING signalling during RNA virus infection. Cytokine & Growth Factor Reviews. 2014;25(6):669–97. Epub August 24, 2014. 10.1016/j.cytogfr.2014.08.004; PubMed Central PMCID: PMC4330990 25212897 PMC4330990

[37] Alessandra Zevini DO, Hiscott J. Crosstalk between Cytoplasmic RIG-I and STING Sensing Pathways. Trends in Immunology. 2017;38(3):194–205. 10.1016/j.it.2016.12.004 PubMed Central PMCID: PMC5329138 28073693 PMC5329138

[38] Aguirre S, Luthra P, Sanchez-Aparicio MT, Maestre AM, Patel J, Lamothe F, et al Dengue virus NS2B protein targets cGAS for degradation and prevents mitochondrial DNA sensing during infection. Nat Microbiol. 2017;2:17037 Epub 2017/03/28. 10.1038/nmicrobiol.2017.37 . 28346446 PMC7457382

[39] Aguirre S, Maestre AM, Pagni S, Patel JR, Savage T, Gutman D, et al DENV inhibits type-I IFN production in infected cells by cleaving human STING. PLoS Pathog. 2012;8(10):e1002934 Epub 2012/10/12. 10.1371/journal.ppat.1002934 23055924 PMC3464218

[40] Franz KM, Neidermyer WJ, Tan YJ, Whelan SPJ, Kagan JC. STING-dependent translation inhibition restricts RNA virus replication. Proc Natl Acad Sci U S A. 2018;115(9):E2058–E67. Epub 2018/02/15. 10.1073/pnas.1716937115 29440426 PMC5834695

[41] Ishikawa H, Barber GN. STING is an endoplasmic reticulum adaptor that facilitates innate immune signalling. Nature. 2008;455(7213):674–8. Epub 2008/08/30. 10.1038/nature07317 18724357 PMC2804933

[42] Ishikawa H, Ma Z, Barber GN. STING regulates intracellular DNA-mediated, type I interferon-dependent innate immunity. Nature. 2009;461(7265):788–92. Epub 2009/09/25. 10.1038/nature08476 19776740 PMC4664154

[43] Nazmi A, Mukhopadhyay R, Dutta K, Basu A. STING mediates neuronal innate immune response following Japanese encephalitis virus infection. Sci Rep. 2012;2:347 Epub 2012/04/04. 10.1038/srep00347 22470840 PMC3317237

[44] Nitta S, Sakamoto N, Nakagawa M, Kakinuma S, Mishima K, Kusano-Kitazume A, et al Hepatitis C virus NS4B protein targets STING and abrogates RIG-I-mediated type I interferon-dependent innate immunity. Hepatology. 2013;57(1):46–58. Epub 2012/08/23. 10.1002/hep.26017 . 22911572

[45] Sun L, Xing Y, Chen X, Zheng Y, Yang Y, Nichols DB, et al Coronavirus papain-like proteases negatively regulate antiviral innate immune response through disruption of STING-mediated signaling. PLoS One. 2012;7(2):e30802 Epub 2012/02/09. 10.1371/journal.pone.0030802 22312431 PMC3270028

[46] Yu CY, Chang TH, Liang JJ, Chiang RL, Lee YL, Liao CL, et al Dengue virus targets the adaptor protein MITA to subvert host innate immunity. PLoS Pathog. 2012;8(6):e1002780 Epub 2012/07/05. 10.1371/journal.ppat.1002780 22761576 PMC3386177

[47] Sun L, Wu J, Du F, Chen X, Chen ZJ. Cyclic GMP-AMP synthase is a cytosolic DNA sensor that activates the type I interferon pathway. Science. 2013;339(6121):786–91. Epub 2012/12/22. 10.1126/science.1232458 23258413 PMC3863629

[48] Chen Q, Sun L, Chen ZJ. Regulation and function of the cGAS–STING pathway of cytosolic DNA sensing. Nature Immunology. 2016;17:1142 10.1038/ni.3558 27648547

[49] Jin L, Waterman PM, Jonscher KR, Short CM, Reisdorph NA, Cambier JC. MPYS, a novel membrane tetraspanner, is associated with major histocompatibility complex class II and mediates transduction of apoptotic signals. Mol Cell Biol. 2008;28(16):5014–26. Epub 2008/06/19. 10.1128/MCB.00640-08 18559423 PMC2519703

[50] Mukai K, Konno H, Akiba T, Uemura T, Waguri S, Kobayashi T, et al Activation of STING requires palmitoylation at the Golgi. Nat Commun. 2016;7:11932 Epub 2016/06/22. 10.1038/ncomms11932 27324217 PMC4919521

[51] Sourisseau M, Schilte C, Casartelli N, Trouillet C, Guivel-Benhassine F, Rudnicka D, et al Characterization of reemerging chikungunya virus. PLoS Pathog. 2007;3(6):e89 Epub 2007/07/03. 10.1371/journal.ppat.0030089 17604450 PMC1904475

[52] Fernandez-Sesma A, Marukian S, Ebersole BJ, Kaminski D, Park MS, Yuen T, et al Influenza virus evades innate and adaptive immunity via the NS1 protein. J Virol. 2006;80(13):6295–304. Epub 2006/06/16. 10.1128/JVI.02381-05 16775317 PMC1488970

[53] Park MS, Garcia-Sastre A, Cros JF, Basler CF, Palese P. Newcastle disease virus V protein is a determinant of host range restriction. J Virol. 2003;77(17):9522–32. Epub 2003/08/14. 10.1128/jvi.77.17.9522-9532.2003 12915566 PMC187425

[54] Dai P, Wang W, Cao H, Avogadri F, Dai L, Drexler I, et al Modified vaccinia virus Ankara triggers type-I IFN production in murine conventional dendritic cells via a cGAS/STING-mediated cytosolic DNA-sensing pathway. PLoS Pathog. 2014;10(4):e1003989 Epub 2014/04/20. 10.1371/journal.ppat.1003989 24743339 PMC3990710

[55] Aguirre S, Fernandez-Sesma A. Collateral Damage during Dengue Virus Infection: Making Sense of DNA by cGAS. J Virol. 2017;91(14). Epub 2017/04/28. 10.1128/JVI.01081-16 28446670 PMC5487551

[56] Joubert PE, Werneke SW, de la Calle C, Guivel-Benhassine F, Giodini A, Peduto L, et al Chikungunya virus-induced autophagy delays caspase-dependent cell death. J Exp Med. 2012;209(5):1029–47. Epub 2012/04/18. 10.1084/jem.20110996 22508836 PMC3348111

[57] Sauer JD, Sotelo-Troha K, von Moltke J, Monroe KM, Rae CS, Brubaker SW, et al The N-ethyl-N-nitrosourea-induced Goldenticket mouse mutant reveals an essential function of Sting in the in vivo interferon response to Listeria monocytogenes and cyclic dinucleotides. Infect Immun. 2011;79(2):688–94. Epub 2010/11/26. 10.1128/IAI.00999-10 21098106 PMC3028833

[58] Schmidt EK, Clavarino G, Ceppi M, Pierre P. SUnSET, a nonradioactive method to monitor protein synthesis. Nat Methods. 2009;6(4):275–7. Epub 2009/03/24. 10.1038/nmeth.1314 . 19305406

[59] Krejbich-Trotot P, Gay B, Li-Pat-Yuen G, Hoarau JJ, Jaffar-Bandjee MC, Briant L, et al Chikungunya triggers an autophagic process which promotes viral replication. Virol J. 2011;8:432 Epub 2011/09/10. 10.1186/1743-422X-8-432 21902836 PMC3179960

[60] Heckmann BL, Yang X, Zhang X, Liu J. The autophagic inhibitor 3-methyladenine potently stimulates PKA-dependent lipolysis in adipocytes. Br J Pharmacol. 2013;168(1):163–71. Epub 2012/07/24. 10.1111/j.1476-5381.2012.02110.x 22817685 PMC3570012

[61] Dai S, Wang B, Li W, Wang L, Song X, Guo C, et al Systemic application of 3-methyladenine markedly inhibited atherosclerotic lesion in ApoE(-/-) mice by modulating autophagy, foam cell formation and immune-negative molecules. Cell Death Dis. 2016;7(12):e2498 Epub 2016/12/03. 10.1038/cddis.2016.376 27906187 PMC5260998

[62] Wu YT, Tan HL, Shui G, Bauvy C, Huang Q, Wenk MR, et al Dual role of 3-methyladenine in modulation of autophagy via different temporal patterns of inhibition on class I and III phosphoinositide 3-kinase. J Biol Chem. 2010;285(14):10850–61. Epub 2010/02/04. 10.1074/jbc.M109.080796 20123989 PMC2856291

[63] Tanida I, Mizushima N, Kiyooka M, Ohsumi M, Ueno T, Ohsumi Y, et al Apg7p/Cvt2p: A novel protein-activating enzyme essential for autophagy. Mol Biol Cell. 1999;10(5):1367–79. Epub 1999/05/08. 10.1091/mbc.10.5.1367 10233150 PMC25280

[64] Ouyang S, Song X, Wang Y, Ru H, Shaw N, Jiang Y, et al Structural analysis of the STING adaptor protein reveals a hydrophobic dimer interface and mode of cyclic di-GMP binding. Immunity. 2012;36(6):1073–86. Epub 2012/05/15. 10.1016/j.immuni.2012.03.019 22579474 PMC3654694

[65] Wu X, Wu FH, Wang X, Wang L, Siedow JN, Zhang W, et al Molecular evolutionary and structural analysis of the cytosolic DNA sensor cGAS and STING. Nucleic Acids Res. 2014;42(13):8243–57. Epub 2014/07/02. 10.1093/nar/gku569 24981511 PMC4117786

[66] Laakkonen P, Ahola T, Kaariainen L. The effects of palmitoylation on membrane association of Semliki forest virus RNA capping enzyme. J Biol Chem. 1996;271(45):28567–71. Epub 1996/11/08. 10.1074/jbc.271.45.28567 . 8910486

[67] Rotem Z, Cox RA, Isaacs A. Inhibition of virus multiplication by foreign nucleic acid. Nature. 1963;197:564–6. Epub 1963/02/09. 10.1038/197564a0 . 13975288

[68] Stetson DB, Medzhitov R. Recognition of cytosolic DNA activates an IRF3-dependent innate immune response. Immunity. 2006;24(1):93–103. Epub 2006/01/18. 10.1016/j.immuni.2005.12.003 . 16413926

[69] Civril F, Deimling T, de Oliveira Mann CC, Ablasser A, Moldt M, Witte G, et al Structural mechanism of cytosolic DNA sensing by cGAS. Nature. 2013;498(7454):332–7. Epub 2013/06/01. 10.1038/nature12305 23722159 PMC3768140

[70] Cai X, Chiu YH, Chen ZJ. The cGAS-cGAMP-STING pathway of cytosolic DNA sensing and signaling. Mol Cell. 2014;54(2):289–96. Epub 2014/04/29. 10.1016/j.molcel.2014.03.040 . 24766893

[71] Judith D, Mostowy S, Bourai M, Gangneux N, Lelek M, Lucas-Hourani M, et al Species-specific impact of the autophagy machinery on Chikungunya virus infection. EMBO Rep. 2013;14(6):534–44. Epub 2013/04/27. 10.1038/embor.2013.51 23619093 PMC3674439

[72] Shoji-Kawata S, Sumpter R, Leveno M, Campbell GR, Zou Z, Kinch L, et al Identification of a candidate therapeutic autophagy-inducing peptide. Nature. 2013;494(7436):201–6. Epub 2013/02/01. 10.1038/nature11866 23364696 PMC3788641

[73] Holm CK, Jensen SB, Jakobsen MR, Cheshenko N, Horan KA, Moeller HB, et al Virus-cell fusion as a trigger of innate immunity dependent on the adaptor STING. Nat Immunol. 2012;13(8):737–43. Epub 2012/06/19. 10.1038/ni.2350 22706339 PMC3411909

[74] Holm CK, Rahbek SH, Gad HH, Bak RO, Jakobsen MR, Jiang Z, et al Influenza A virus targets a cGAS-independent STING pathway that controls enveloped RNA viruses. Nat Commun. 2016;7:10680 Epub 2016/02/20. 10.1038/ncomms10680 26893169 PMC4762884

[75] de Groot RJ, Rumenapf T, Kuhn RJ, Strauss EG, Strauss JH. Sindbis virus RNA polymerase is degraded by the N-end rule pathway. Proc Natl Acad Sci U S A. 1991;88(20):8967–71. Epub 1991/10/15. 10.1073/pnas.88.20.8967 1924357 PMC52632

[76] Fata CL, Sawicki SG, Sawicki DL. Modification of Asn374 of nsP1 suppresses a Sindbis virus nsP4 minus-strand polymerase mutant. J Virol. 2002;76(17):8641–9. Epub 2002/08/07. 10.1128/jvi.76.17.8641-8649.2002 12163583 PMC136982

[77] Rupp JC, Jundt N, Hardy RW. Requirement for the amino-terminal domain of sindbis virus nsP4 during virus infection. J Virol. 2011;85(7):3449–60. Epub 2011/01/21. 10.1128/JVI.02058-10 21248049 PMC3067876

[78] Shirako Y, Strauss EG, Strauss JH. Suppressor mutations that allow sindbis virus RNA polymerase to function with nonaromatic amino acids at the N-terminus: evidence for interaction between nsP1 and nsP4 in minus-strand RNA synthesis. Virology. 2000;276(1):148–60. Epub 2000/10/07. 10.1006/viro.2000.0544 . 11022003

[79] Nishida Y, Arakawa S, Fujitani K, Yamaguchi H, Mizuta T, Kanaseki T, et al Discovery of Atg5/Atg7-independent alternative macroautophagy. Nature. 2009;461(7264):654–8. Epub 2009/10/02. 10.1038/nature08455 . 19794493

[80] Chen M, Meng Q, Qin Y, Liang P, Tan P, He L, et al TRIM14 Inhibits cGAS Degradation Mediated by Selective Autophagy Receptor p62 to Promote Innate Immune Responses. Mol Cell. 2016;64(1):105–19. Epub 2016/09/27. 10.1016/j.molcel.2016.08.025 . 27666593

[81] Ahola T, Lampio A, Auvinen P, Kaariainen L. Semliki Forest virus mRNA capping enzyme requires association with anionic membrane phospholipids for activity. EMBO J. 1999;18(11):3164–72. Epub 1999/06/05. 10.1093/emboj/18.11.3164 10357827 PMC1171397

[82] Kumar S, Kumar A, Mamidi P, Tiwari A, Kumar S, Mayavannan A, et al Chikungunya virus nsP1 interacts directly with nsP2 and modulates its ATPase activity. Sci Rep. 2018;8(1):1045 Epub 2018/01/20. 10.1038/s41598-018-19295-0 29348627 PMC5773547

[83] Lampio A, Kilpelainen I, Pesonen S, Karhi K, Auvinen P, Somerharju P, et al Membrane binding mechanism of an RNA virus-capping enzyme. J Biol Chem. 2000;275(48):37853–9. Epub 2000/09/14. 10.1074/jbc.M004865200 . 10984480

[84] Zhong B, Zhang L, Lei C, Li Y, Mao AP, Yang Y, et al The ubiquitin ligase RNF5 regulates antiviral responses by mediating degradation of the adaptor protein MITA. Immunity. 2009;30(3):397–407. Epub 2009/03/17. 10.1016/j.immuni.2009.01.008 . 19285439

[85] Zhong B, Yang Y, Li S, Wang YY, Li Y, Diao F, et al The adaptor protein MITA links virus-sensing receptors to IRF3 transcription factor activation. Immunity. 2008;29(4):538–50. Epub 2008/09/27. 10.1016/j.immuni.2008.09.003 . 18818105

[86] Delang L, Li C, Tas A, Querat G, Albulescu IC, De Burghgraeve T, et al The viral capping enzyme nsP1: a novel target for the inhibition of chikungunya virus infection. Sci Rep. 2016;6:31819 Epub 2016/08/23. 10.1038/srep31819 27545976 PMC4992889

[87] Chen X, Yang X, Zheng Y, Yang Y, Xing Y, Chen Z. SARS coronavirus papain-like protease inhibits the type I interferon signaling pathway through interaction with the STING-TRAF3-TBK1 complex. Protein Cell. 2014;5(5):369–81. Epub 2014/03/14. 10.1007/s13238-014-0026-3 24622840 PMC3996160

[88] Ding Q, Cao X, Lu J, Huang B, Liu YJ, Kato N, et al Hepatitis C virus NS4B blocks the interaction of STING and TBK1 to evade host innate immunity. J Hepatol. 2013;59(1):52–8. Epub 2013/04/02. 10.1016/j.jhep.2013.03.019 . 23542348

[89] Rodriguez-Madoz JR, Belicha-Villanueva A, Bernal-Rubio D, Ashour J, Ayllon J, Fernandez-Sesma A. Inhibition of the type I interferon response in human dendritic cells by dengue virus infection requires a catalytically active NS2B3 complex. J Virol. 2010;84(19):9760–74. Epub 2010/07/28. 10.1128/JVI.01051-10 20660196 PMC2937777

[90] Levitt NH, Ramsburg HH, Hasty SE, Repik PM, Cole FE Jr., Lupton HW. Development of an attenuated strain of chikungunya virus for use in vaccine production. Vaccine. 1986;4(3):157–62. Epub 1986/09/01. 10.1016/0264-410x(86)90003-4 . 3020820

[91] Diamond MS, Edgil D, Roberts TG, Lu B, Harris E. Infection of human cells by dengue virus is modulated by different cell types and viral strains. J Virol. 2000;74(17):7814–23. Epub 2000/08/10. 10.1128/jvi.74.17.7814-7823.2000 10933688 PMC112311

[92] Rodriguez-Madoz JR, Bernal-Rubio D, Kaminski D, Boyd K, Fernandez-Sesma A. Dengue virus inhibits the production of type I interferon in primary human dendritic cells. J Virol. 2010;84(9):4845–50. Epub 2010/02/19. 10.1128/JVI.02514-09 20164230 PMC2863727

[93] Park MS, Shaw ML, Munoz-Jordan J, Cros JF, Nakaya T, Bouvier N, et al Newcastle disease virus (NDV)-based assay demonstrates interferon-antagonist activity for the NDV V protein and the Nipah virus V, W, and C proteins. J Virol. 2003;77(2):1501–11. Epub 2002/12/28. 10.1128/jvi.77.2.1501-1511.2003 12502864 PMC140815

[94] Diamond MS, Roberts TG, Edgil D, Lu B, Ernst J, Harris E. Modulation of Dengue virus infection in human cells by alpha, beta, and gamma interferons. J Virol. 2000;74(11):4957–66. Epub 2000/05/09. 10.1128/jvi.74.11.4957-4966.2000 10799569 PMC110847

[95] Yount JS, Moltedo B, Yang YY, Charron G, Moran TM, Lopez CB, et al Palmitoylome profiling reveals S-palmitoylation-dependent antiviral activity of IFITM3. Nat Chem Biol. 2010;6(8):610–4. Epub 2010/07/06. 10.1038/nchembio.405 20601941 PMC2928251

[96] Chesarino NM, Hach JC, Chen JL, Zaro BW, Rajaram MV, Turner J, et al Chemoproteomics reveals Toll-like receptor fatty acylation. BMC Biol. 2014;12:91 Epub 2014/11/06. 10.1186/s12915-014-0091-3 25371237 PMC4240870

[97] McMichael TM, Zhang L, Chemudupati M, Hach JC, Kenney AD, Hang HC, et al The palmitoyltransferase ZDHHC20 enhances interferon-induced transmembrane protein 3 (IFITM3) palmitoylation and antiviral activity. J Biol Chem. 2017;292(52):21517–26. Epub 2017/10/29. 10.1074/jbc.M117.800482 29079573 PMC5766958

