# Supplementary figures and images for: Chikungunya virus antagonizes cGAS-STING mediated type-I interferon responses by degrading cGAS
Source: PLoS Pathog. 2020 Oct 15;16(10):e1008999. doi: 10.1371/journal.ppat.1008999 (PMC7591055; doi:10.1371/journal.ppat.1008999)

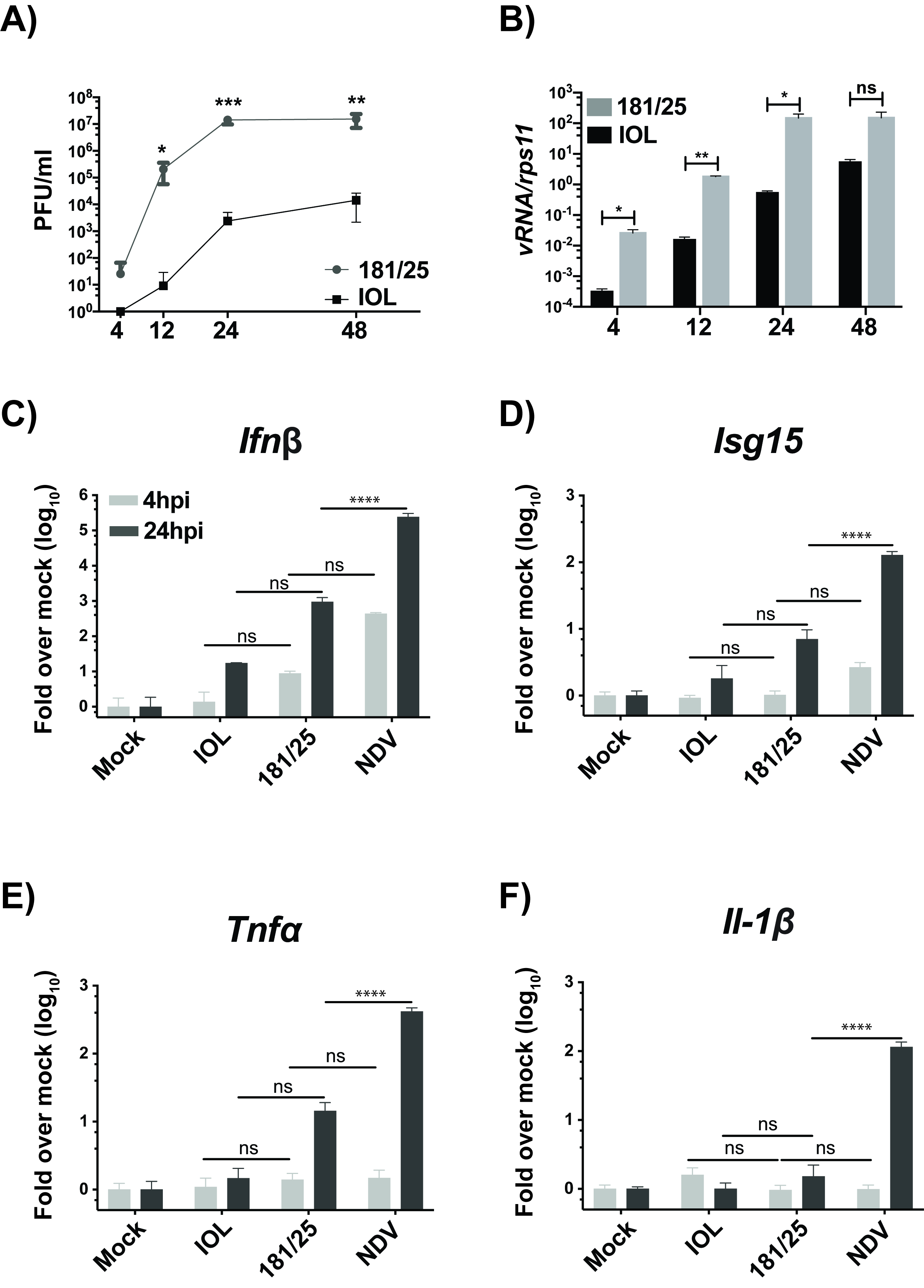

Supplement: S1 Fig — Human foreskin fibroblasts (HFF-1) were either mock infected or infected with CHIKV IOL, CHIKV 181/25, or NDVB1 at an MOI of 0.1. Supernatants and RNA were collected 4, 12, 24, and 48 hpi. (A) Plaque assay on BHKs of supernatants from infected HFF-1s. (B) RT-qPCR analysis of infected cells measuring nsP2. (C-F) RT-qPCR analysis of innate immune transcripts in infected cells at 4 and 24 hpi with primers specific for Ifnß, Isg15, Tnf-a, and Il-1ß. Data shown representative of two independent experiments. All RT-qPCR genes represented in this figure were normalized to rps11 then represented as vRNA/rps11 or fold over mock as indicated. Data are represented as means ± SD (n = 3). Statistical analysis was done by student’s t test (A & B) or two way ANOVA with Tukey’s multiple comparisons (C-F) (ns = not significant, * = p<0.05, ** = p<0.01, *** = p<0.001, **** = p<0.0001). (TIF) [file ppat.1008999.s001.tif]

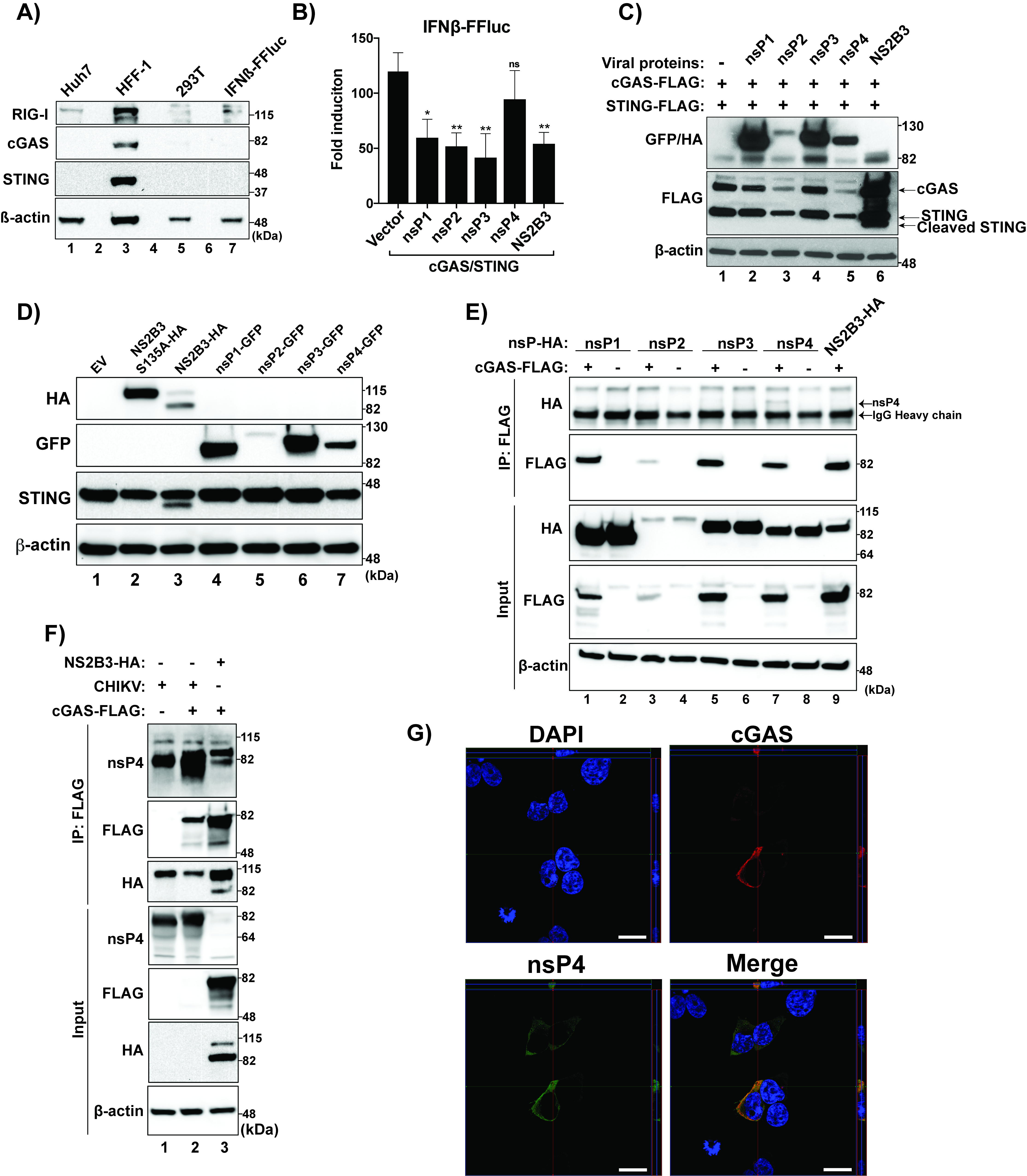

Supplement: S2 Fig — (A) Human liver cells (Huh7) which do not endogenously express cGAS or STING, human foreskin fibroblasts (HFF-1), human embryonic kidney (HEK-293T), and HEK-293T cells stably expressing an IFNβ promoter driving the production of firefly luciferase (293T-IFNβ-FFluc) were lysed and protein was analyzed via SDS-PAGE. Expression levels of endogenous cGAS, STING, and RIG-I are shown for the respective cell types. Data representative of two independent experiments. (B) 293T-IFNb-FFluc cells were transfected with cGAS and STING in conjunction with empty vector (vector), or the indicated viral proteins (nsPs 1–4 of CHIKV-RT). Cells were allowed to rest for 36hrs before lysis for collection of protein or quantification of luminescence. (C) Input protein expression for reporter experiment (B) was visualized via SDS-PAGE followed by immunoblotting. Data representative of four independent experiments. Data are represented by means ± SD (n = 3), fold induction over mock. Statistical analysis was done with student’s t tests (* = p<0.05, ** = p<0.01, *** = p<0.001). (D) HEK-293T cells were transfected with indicated constructs and cells were lysed 24 hpt. DENV-2 NS2B3 served as a positive control for STING cleavage/degradation while the catalytically inactive NS2B3 S135A was used as a negative control. GFP tagged CHIKV-RT nsP constructs were used to test for degradation or cleavage of STING. Protein lysates were analyzed via SDS-PAGE and subsequent immunoblotting. Data representative of one independent experiment. (E) HEK-293T cells were transfected with indicated constructs (nsPs 1-4-HA CHIKV-RT) and cells were allowed to rest for 24 hrs before lysis. Lysates were subjected to immunoprecipitation against a Flag epitope and proteins were visualized via SDS-PAGE and immunoblotting. Data representative of three independent experiments. (F) Indicated constructs were expressed in 293T cells and cells were allowed to rest for 16 hrs. After resting, cells were infected with either mock [file ppat.1008999.s002.tif]

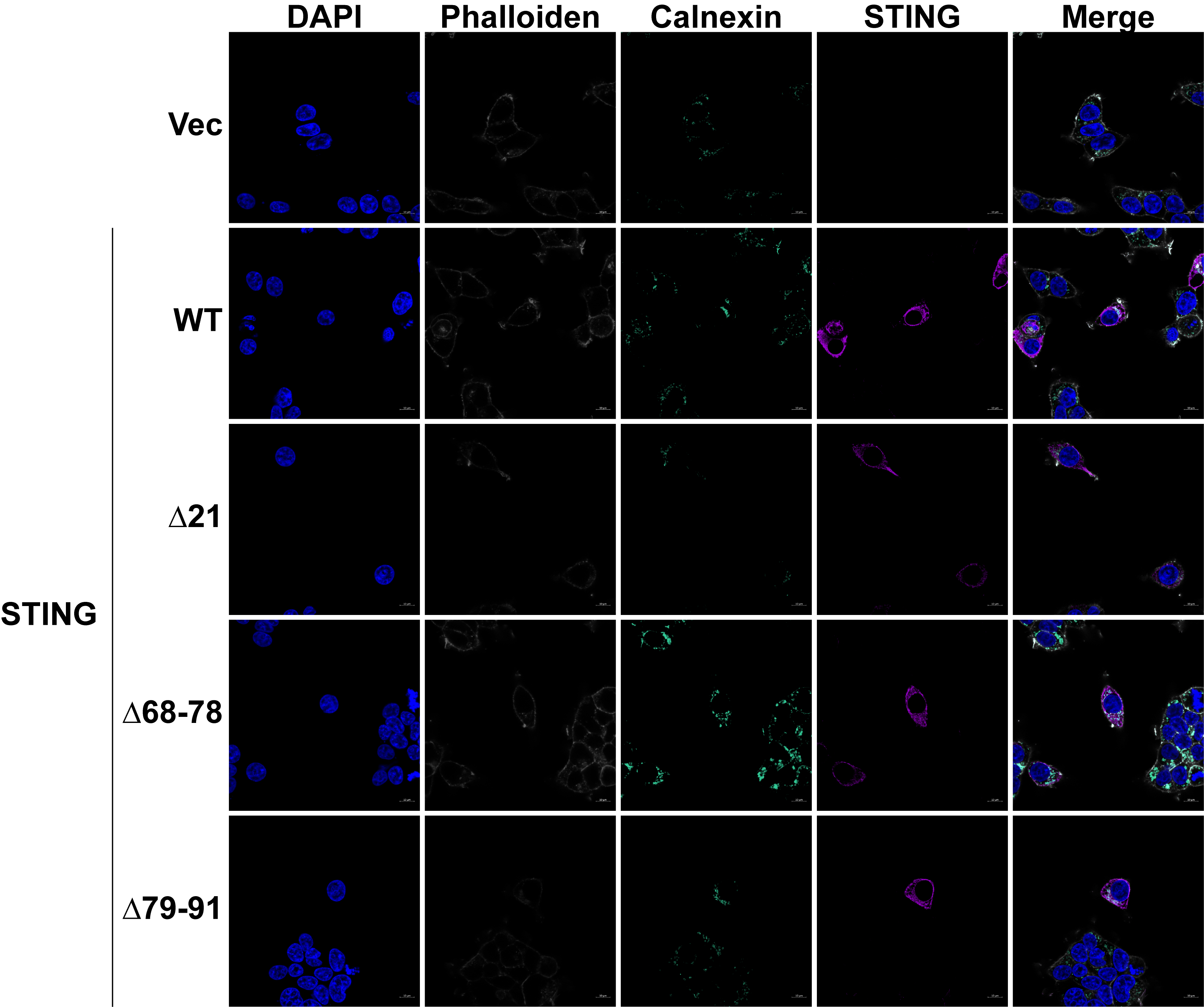

Supplement: S3 Fig — HEK 293Ts were transfected with empty vector or indicated STING constructs. 24 hpi cells were fixed with 4% formaldehyde and permeabilized with 0.01% triton-X before staining with antibodies specific for Flag-(STING: purple), Calnexin (aqua), Phalloiden (grey), or DAPI (blue). Cells were visualized using a Zeiss LSM 880 with Airyscan. Scale bars = 10um. Data are representative of two independent experiments. (TIF) [file ppat.1008999.s003.tif]

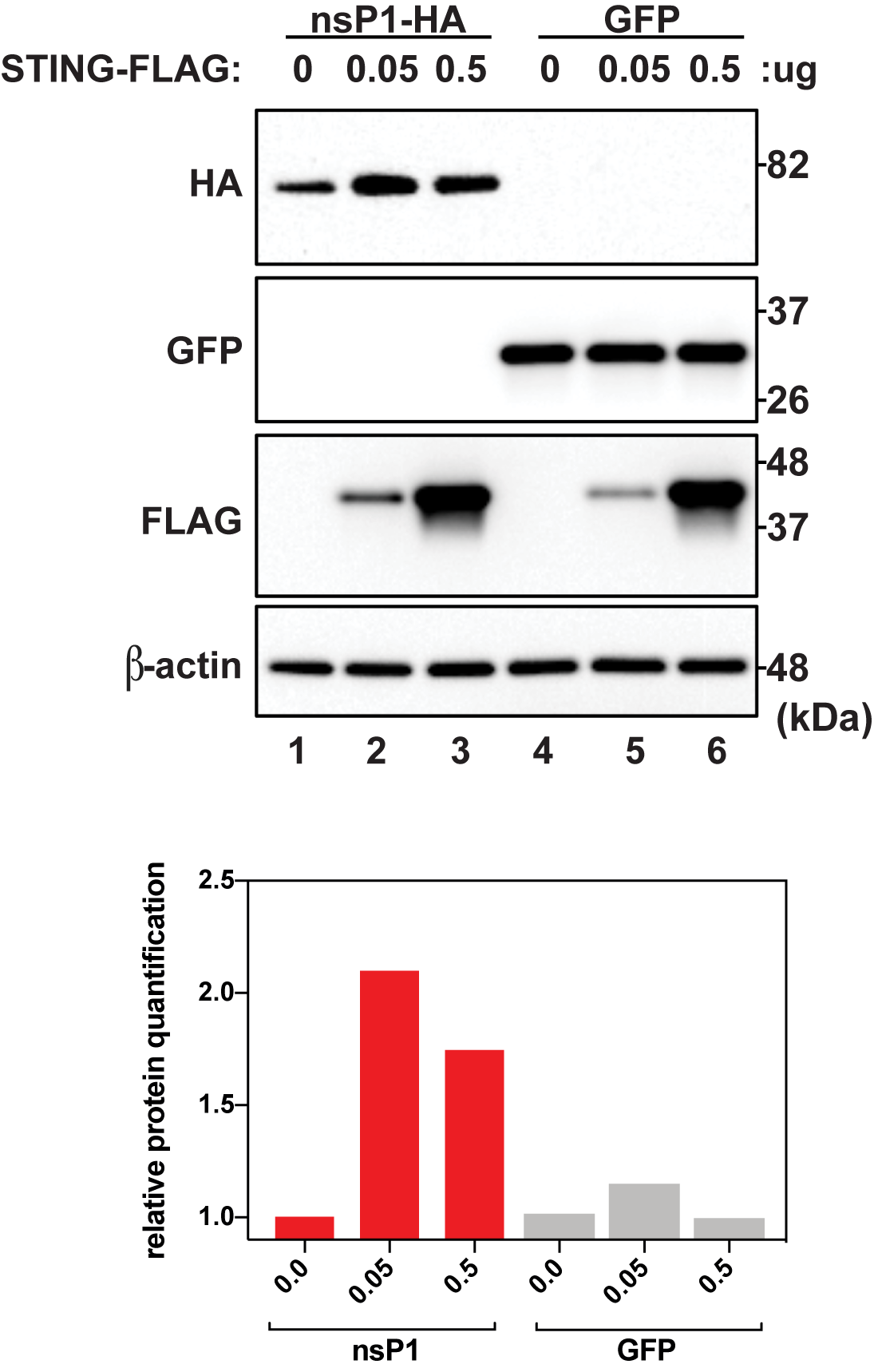

Supplement: S4 Fig — HEK-293T cells were transfected with increasing concentrations of the indicated constructs and cells were lysed 24 hpt. Lysates were analyzed via SDS-PAGE and immunoblotting. Densitometry measurement of bands in figure blot performed with ImageJ software. Data are representative of two independent experiments. (TIF) [file ppat.1008999.s004.tif]
